# Genetic and Genomic Architecture of the Evolution of Resistance to Antifungal Drug Combinations

**DOI:** 10.1371/journal.pgen.1003390

**Published:** 2013-04-04

**Authors:** Jessica A. Hill, Ron Ammar, Dax Torti, Corey Nislow, Leah E. Cowen

**Affiliations:** 1 Department of Molecular Genetics, University of Toronto, Toronto, Ontario, Canada; 2 Donnelly Centre for Cellular and Biomolecular Research, University of Toronto, Toronto, Ontario, Canada; 3 Donnelly Sequencing Centre, University of Toronto, Toronto, Ontario, Canada; Washington University School of Medicine, United States of America

## Abstract

The evolution of drug resistance in fungal pathogens compromises the efficacy of the limited number of antifungal drugs. Drug combinations have emerged as a powerful strategy to enhance antifungal efficacy and abrogate drug resistance, but the impact on the evolution of drug resistance remains largely unexplored. Targeting the molecular chaperone Hsp90 or its downstream effector, the protein phosphatase calcineurin, abrogates resistance to the most widely deployed antifungals, the azoles, which inhibit ergosterol biosynthesis. Here, we evolved experimental populations of the model yeast *Saccharomyces cerevisiae* and the leading human fungal pathogen *Candida albicans* with azole and an inhibitor of Hsp90, geldanamycin, or calcineurin, FK506. To recapitulate a clinical context where Hsp90 or calcineurin inhibitors could be utilized in combination with azoles to render resistant pathogens responsive to treatment, the evolution experiment was initiated with strains that are resistant to azoles in a manner that depends on Hsp90 and calcineurin. Of the 290 lineages initiated, most went extinct, yet 14 evolved resistance to the drug combination. Drug target mutations that conferred resistance to geldanamycin or FK506 were identified and validated in five evolved lineages. Whole-genome sequencing identified mutations in a gene encoding a transcriptional activator of drug efflux pumps, *PDR1*, and a gene encoding a transcriptional repressor of ergosterol biosynthesis genes, *MOT3*, that transformed azole resistance of two lineages from dependent on calcineurin to independent of this regulator. Resistance also arose by mutation that truncated the catalytic subunit of calcineurin, and by mutation in *LCB1*, encoding a sphingolipid biosynthetic enzyme. Genome analysis revealed extensive aneuploidy in four of the *C. albicans* lineages. Thus, we identify molecular determinants of the transition of azole resistance from calcineurin dependence to independence and establish multiple mechanisms by which resistance to drug combinations evolves, providing a foundation for predicting and preventing the evolution of drug resistance.

## Author Summary

Fungal infections are a leading cause of mortality worldwide and are difficult to treat due to the limited number of antifungal drugs, whose effectiveness is compromised by the emergence of drug resistance. A powerful strategy to combat drug resistance is combination therapy. Inhibiting the molecular chaperone Hsp90 or its downstream effector calcineurin cripples fungal stress responses and abrogates drug resistance. Here we provide the first analysis of the genetic and genomic changes that underpin the evolution of resistance to antifungal drug combinations in the leading human fungal pathogen, *Candida albicans*, and model yeast, *Saccharomyces cerevisiae*. We evolved experimental populations with combinations of inhibitors of Hsp90 or calcineurin and the most widely used antifungal in the clinic, the azoles, which inhibit ergosterol biosynthesis. We harnessed whole-genome sequencing to identify diverse resistance mutations among the 14 lineages that evolved resistance to the drug combination. These included mutations in genes encoding the drug targets, a transcriptional regulator of multidrug transporters, a transcriptional repressor of ergosterol biosynthesis enzymes, and a regulator of sphingolipid biosynthesis. We also identified extensive aneuploidies in several *C. albicans* lineages. Our study reveals multiple mechanisms by which resistance to drug combination can evolve, suggesting new strategies to combat drug resistance.

## Introduction

The evolution of drug resistance is a ubiquitous phenomenon that has a profound impact on human health. With the widespread deployment of antimicrobial agents in both clinical and environmental settings, the rate at which resistance evolves in pathogen populations far outpaces the rate at which new drugs are developed [1], [2]. Drug resistance threatens the utility of the limited arsenal of antimicrobial agents. The economic costs are staggering and exceed $33 billion in the United States alone to cover treatment of drug-resistant infections in patients, eradication of resistant pathogens in agriculture, and crop losses to resistant pests [3]. The evolution of resistance to antifungal drugs is of particular concern given the increasing incidence of life-threatening invasive fungal infections, and the limited number of antifungal drugs with distinct targets [4]. Unlike for antibacterials, fungal-specific drug targets are limited, in part due to the close evolutionary relationships of these eukaryotic pathogens with their human hosts, rendering most treatments toxic to the host or ineffective in combating infections [5]. Even with current treatment options, mortality rates due to invasive fungal infections often exceed 50%, and fungal pathogens kill as many people as tuberculosis or malaria [6], [7]. Thus, there is a pressing need to develop new strategies to enhance the efficacy of antifungal drugs and to minimize the emergence of drug resistance.

A powerful strategy to extend the life of current antimicrobial agents is drug combination therapy [8]. Combination therapy has the potential to minimize the evolution of drug resistance by more effectively eradicating pathogen populations and by requiring multiple mutations to confer drug resistance [9]. Great success has been achieved with combination therapy in the treatment of HIV [10]–[12], and it is currently the recommended strategy for treatment of tuberculosis and malaria [13], [14]. Combination therapies have been less well explored in the clinic for fungal pathogens. However, targeting cellular regulators of fungal stress responses has emerged as a promising strategy to enhance the efficacy of antifungal drugs and to abrogate drug resistance [5], [15]. Two key cellular regulators that are critical for orchestrating cellular responses to drug-induced stress are Hsp90 and calcineurin. The molecular chaperone Hsp90 regulates the stability and function of diverse client proteins [16], [17], and controls stress responses required for drug resistance by stabilizing the protein phosphatase calcineurin [16], [18]–[21]. Compromise of Hsp90 or calcineurin function transforms antifungals from fungistatic to fungicidal and enhances the efficacy of antifungals in mammalian models of systemic and biofilm fungal infections [15], [22]–[24], suggesting that combination therapy with azoles and inhibitors of Hsp90 or calcineurin may provide a powerful strategy to treat life-threatening fungal infections.

Targeting fungal stress response regulators holds particular therapeutic promise for enhancing the efficacy of the azoles, which are the class of antifungal drug that has been used most widely in the clinic for decades. Azoles block the production of ergosterol, the major sterol of fungal cell membranes, by inhibition of lanosterol demethylase, Erg11, resulting in a depletion of ergosterol and the accumulation of the toxic sterol intermediate, 14-α-methyl-3,6-diol, produced by Erg3 [25]. The azoles are generally fungistatic, causing inhibition of growth rather than cell death, and thus impose strong selection for resistance on the surviving fungal population [26]; as a consequence, resistance is frequently encountered in the clinic [27]. Azole resistance mechanisms fall into two broad classes: those that block the effect of the drug on the fungal cell and those that allow the cell to tolerate the drug by minimizing its toxicity [5]. The former class of resistance mechanisms includes upregulation of drug efflux pumps [28], or mutation of the azole target that prevents azole binding [29]. The latter class includes loss-of-function mutations in *ERG3*, which encodes a Δ-5,6-desaturase in the ergosterol biosynthesis pathway; Erg3 loss-of-function blocks the accumulation of a toxic sterol intermediate, conferring azole resistance that is contingent on cellular stress responses [16], [30]. Azole resistance acquired by loss of function of Erg3 or by many other mutations is exquisitely dependent on Hsp90 and calcineurin [16]; inhibition of these stress response regulators enhances azole sensitivity of diverse clinical isolates, and compromises azole resistance of isolates that evolved resistance in a human host [16], [18], [23], [31]. Inhibition of Hsp90 or calcineurin with molecules that are well tolerated in humans can impair the evolution of azole resistance [16], [20], though the potential for evolution of resistance to the drug combinations remains unknown.

Azole resistance mechanisms have been studied most extensively in the opportunistic fungal pathogen *Candida albicans* and the model yeast *Saccharomyces cerevisiae*. *C. albicans* is the leading cause of death due to fungal infection [32], and the fourth leading cause of hospital-acquired infectious disease [7], [32]. It is a natural member of the mucosal microbiota of healthy humans, but can cause life-threatening illness in immunocompromised individuals, such as transplant recipients and those infected with HIV [7], [33], [34]. Drug resistance can readily evolve in *C. albicans* in the laboratory and the clinic, and molecular studies have revealed a diversity of resistance mechanisms [35]. Molecular studies with *C. albicans* are hindered by its obligate diploid state, lack of meiotic cycle, unusual codon usage, and inability to maintain plasmids [36], thus complementary experiments are often performed with its genetically tractable relative, *S. cerevisiae*, with which it often shares drug resistance phenotypes and underlying molecular mechanisms [37]. For both species, inhibition of Hsp90 or calcineurin reduces azole resistance acquired by diverse mutations [16], [18], [22], [38]. With short generation times and relatively small genomes, these organisms provide tractable and complementary systems to explore the dynamics and mechanisms underpinning the evolution of resistance to drug combinations.

Here, we provide the first analysis of the genetic and genomic architecture of the evolution of resistance to drug combinations in fungi. To recapitulate a clinical context where Hsp90 or calcineurin inhibitors could be used in combination with azoles to render azole-resistant fungal pathogens responsive to treatment, we initiated an evolution experiment with strains that are resistant to azoles in a manner that depends on Hsp90 and calcineurin. We evolved populations of *S. cerevisiae* and *C. albicans* that were resistant to azoles due to loss of function of Erg3 with a combination of an azole and an inhibitor of Hsp90, geldanamycin, or calcineurin, FK506, to identify the mechanisms by which resistance evolves to the drug combinations. Of 290 lineages initiated, most went extinct, yet 14 evolved resistance. We identified mechanisms of resistance in the evolved lineages using a hypothesis-driven approach based on cross-resistance profiling and a complementary unbiased approach using whole genome sequencing. Resistance mutations in the drug target of FK506 or geldanamycin were identified and validated in five lineages. Non-synonymous substitutions conferring resistance were identified in a transcriptional activator of drug efflux pumps, Pdr1, and in a regulator of sphingolipid biosynthesis, Lcb1. Resistance also arose by premature stop codons in the catalytic subunit of calcineurin and in a repressor of ergosterol biosynthesis genes, Mot3. Several of the mutations conferred resistance to geldanamycin or FK506, while other mutations transformed azole resistance from dependent on calcineurin to independent of this stress response regulator. Genome analysis also identified extensive aneuploidy in four of the *C. albicans* lineages. Thus, we illuminate the molecular basis for the transition of azole resistance from calcineurin dependence to independence, and establish numerous mechanisms by which resistance to drug combinations can evolve, providing a foundation for predicting and preventing the evolution of drug resistance.

## Results

### Experimental evolution of *C. albicans* and *S. cerevisiae* yields resistance to the combination of an azole and an inhibitor of Hsp90 or calcineurin

**Figure 1:**
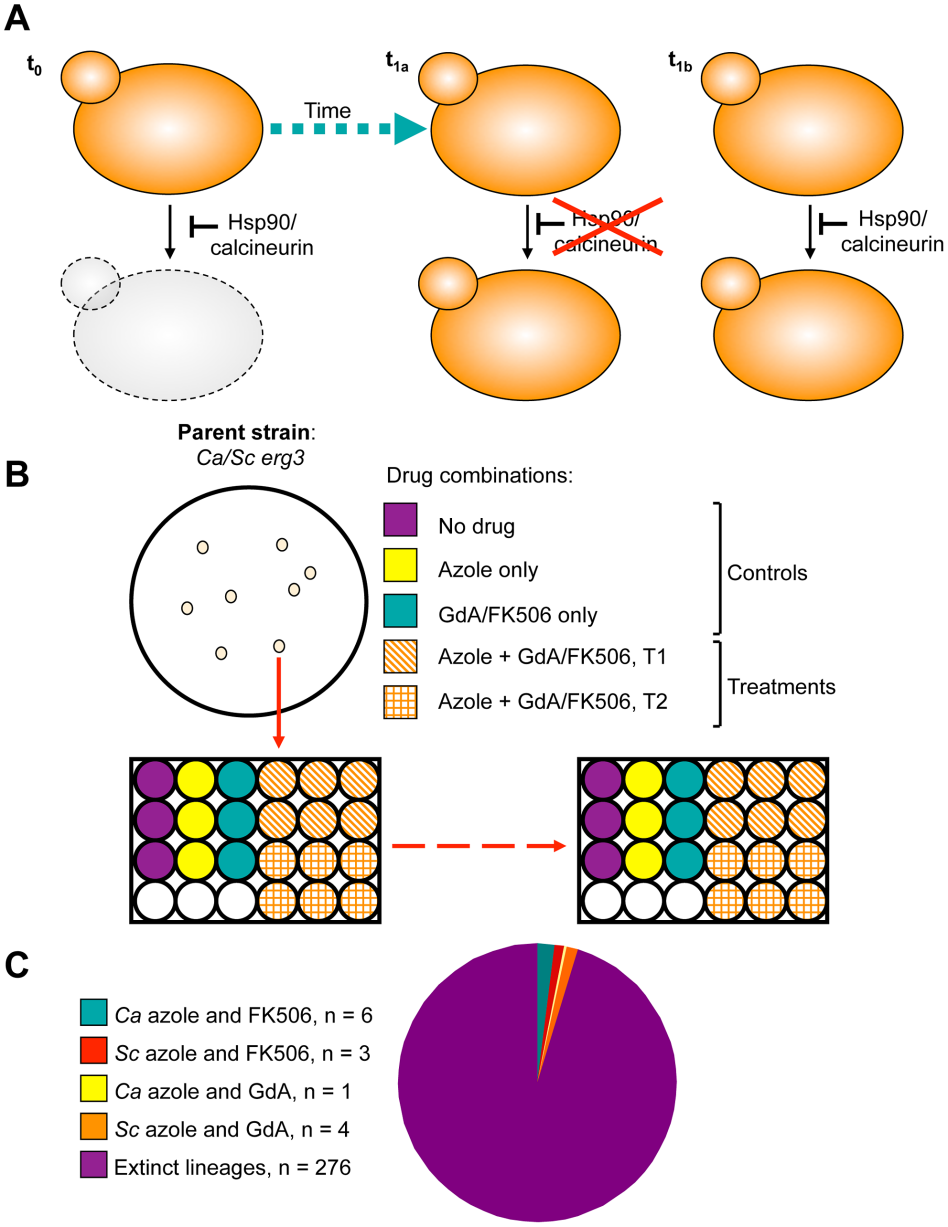
Design and outcome of the experimental evolution of resistance to drug combinations. A) Experimental populations were initiated with *S. cerevisiae* and *C. albicans* strains resistant to azoles due to *erg3* loss of function. This resistance mechanism is contingent on Hsp90 and calcineurin, such that inhibition of either of these cellular stress response regulators results in cell death (**t_0_**). Propagation of these strains in the presence of azole and the Hsp90 inhibitor geldanamycin or azole and the calcineurin inhibitor FK506 at concentrations that exert selection pressure for resistance to the drug combination results in the evolution of resistance to geldanamycin or FK506 (**t_1a_**) or the evolution of an azole resistance mechanism that is independent of Hsp90 or calcineurin (**t_1b_**) among extant lineages. B) Single colony founders were used to initiate evolution experiments in 24- or 96-well plates containing control and treatment wells. Controls consisted of: no drug, azole alone, geldanamycin alone, or FK506 alone, where drug concentrations were not inhibitory. Treatment wells consisted of combinations of azole and geldanamycin or FK506, selected based on dose response matrices (see Figure S2). **C**) Experimental evolution of resistance to azole and geldanamycin or azole and FK506 yielded 14 resistant lineages out of 290 initiated. *Ca* = *Candida albicans; Sc* = *Saccharomyces cerevisiae*.

Inhibition of Hsp90 or calcineurin has emerged as promising strategy to enhance the efficacy of azoles against resistant fungal pathogens, motivating our study to monitor the evolution of resistance to the drug combinations in azole-resistant populations. To do so, we used an experimental evolution approach starting with *C. albicans* and *S. cerevisiae* strains that harbour *erg3* loss-of-function mutations or deletions, rendering them resistant to azoles in a manner that depends on the stress response regulators Hsp90 and calcineurin [5]. Propagation of these strains in the presence of azole and the Hsp90 inhibitor geldanamycin or azole and the calcineurin inhibitor FK506 at concentrations that exert selection pressure for resistance to the drug combination could lead to the evolution of resistance to geldanamycin or FK506, or the evolution of an azole resistance mechanism that is independent of Hsp90 or calcineurin among extant lineages (Figure 1A). Lineages were propagated by serial transfer for between 33 and 100 generations until robust growth in the presence of the drug combination was observed in extant lineages (Figure 1B). The effective population size per lineage was ∼4.6×10^6^, given that cultures reached saturation (∼10^7^ cells/ml) between transfers. Of the 290 lineages initiated, the majority went extinct. Fourteen lineages evolved resistance to the combination of azole and inhibitor of Hsp90 or calcineurin (Figure 1C); seven of these lineages are *C. albicans* and seven are *S. cerevisiae* (Table 1). Six *C. albicans* lineages evolved resistance to azole and FK506 (Ca-F lineages), and only one evolved resistance to azole and geldanamycin (Ca-G lineage). Four *S. cerevisiae* lineages evolved resistance to azole and geldanamycin (Sc-G lineages) and three evolved resistance to azole and FK506 (Sc-F lineages).

**Table 1:** Evolution experiment treatments and conditions.

Strain name	Ancestor	Drug combination evolved in	Fluconazole (FL) or miconazole (M) concentration evolved in (µg/ml)	FK506 or geldanamycin (GdA) concentration evolved in (µM)	Number of transfers (generations)	Number of wells in plate evolved in
Sc-F-1	ScLC7	FL and FK506	32	2.5	13 (∼86)	24
Sc-F-2	ScLC7	FL and FK506	64	0.03	13 (∼86)	24
Sc-F-3	ScLC7	M and FK506	75	0.06	6 (∼40)	24
Ca-F-4	CaLC660	FL and FK506	256	20	9 (∼60)	96
Ca-F-5	CaLC660	FL and FK506	256	20	9 (∼60)	96
Ca-F-6	CaLC660	FL and FK506	256	1.2	13 (∼86)	24
Ca-F-7	CaLC660	FL and FK506	4	2	5 (∼33)	24
Ca-F-8	CaLC660	FL and FK506	4	2	5 (∼33)	24
Ca-F-9	CaLC660	M and FK506	64	1.2	5 (∼33)	24
Ca-F-10	CaLC660	FL and GdA	0.1875	0.16	13 (∼86)	24
Sc-G-11	ScLC10	FL and GdA	256	0.6	13 (∼86)	24
Sc-G-12	ScLC7	FL and GdA	256	0.6	13 (∼86)	24
Sc-G-13	ScLC10	FL and GdA	16	2.5	5 (∼33)	24
Sc-G-14	ScLC10	FL and GdA	16	2.5	5 (∼33)	24

**Figure 2:**
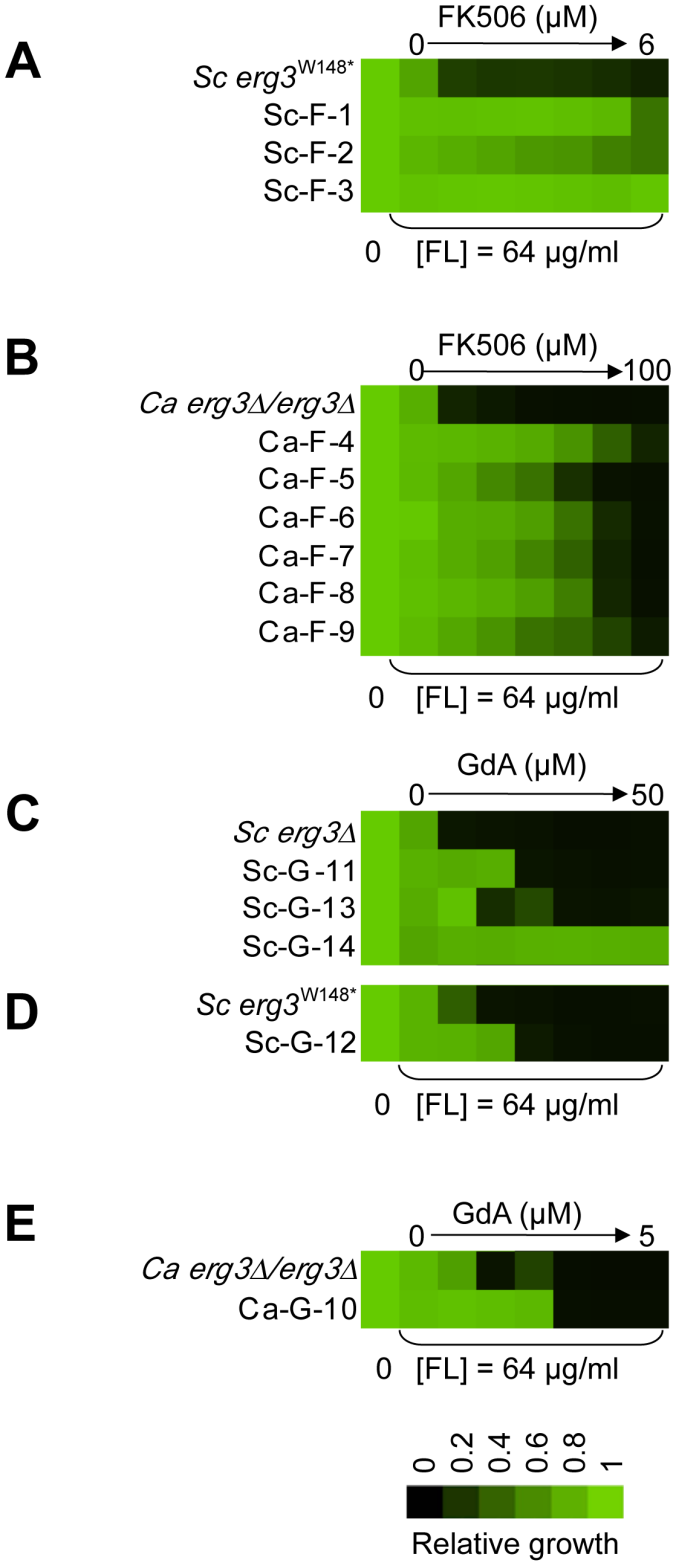
The populations evolved distinct resistance profiles. Levels of resistance to azole and FK506 (A, B) or azole and geldanamycin (C–E) of evolved strains of *S. cerevisiae* (A, C, D) and *C. albicans* (B, E), relative to their ancestors. Resistance was measured with a constant concentration of azole and a gradient of geldanamycin or FK506 in YPD at 30°C for 2 days (B) or 3 days (A, C–E). Optical densities were averaged for duplicate measurements and normalized relative to drug-free controls (see colour bar). GdA = geldanamycin and FL = fluconazole.

Resistance levels to the drug combinations of all fourteen evolved lineages were evaluated by performing minimum inhibitory concentration (MIC) assays in the presence of the inhibitors with which they were evolved, azole and FK506 (Figure 2A and 2B) or azole and geldanamycin (Figure 2C–2E). Because the azole resistance phenotypes of the starting strains were abrogated by geldanamycin or FK506, resistance of the evolved lineages was monitored with a fixed concentration of azole and a gradient of concentrations of geldanamycin or FK506. Resistance was monitored for a population of cells from each archived lineage, and for four clones isolated from the evolved population. In all cases, the clones reflected the resistant phenotype of the population (data not shown), suggestive of strong selective sweeps as mutations were rapidly fixed in the population. For each population, a clone was archived and further analyses were performed on that strain. The lineages evolved distinct levels of resistance to the drug combinations (Figure 2), indicating that they acquired different mutations conferring resistance.

### Cross-resistance assays as a strategy to predict distinct mechanisms of resistance

**Figure 3:**
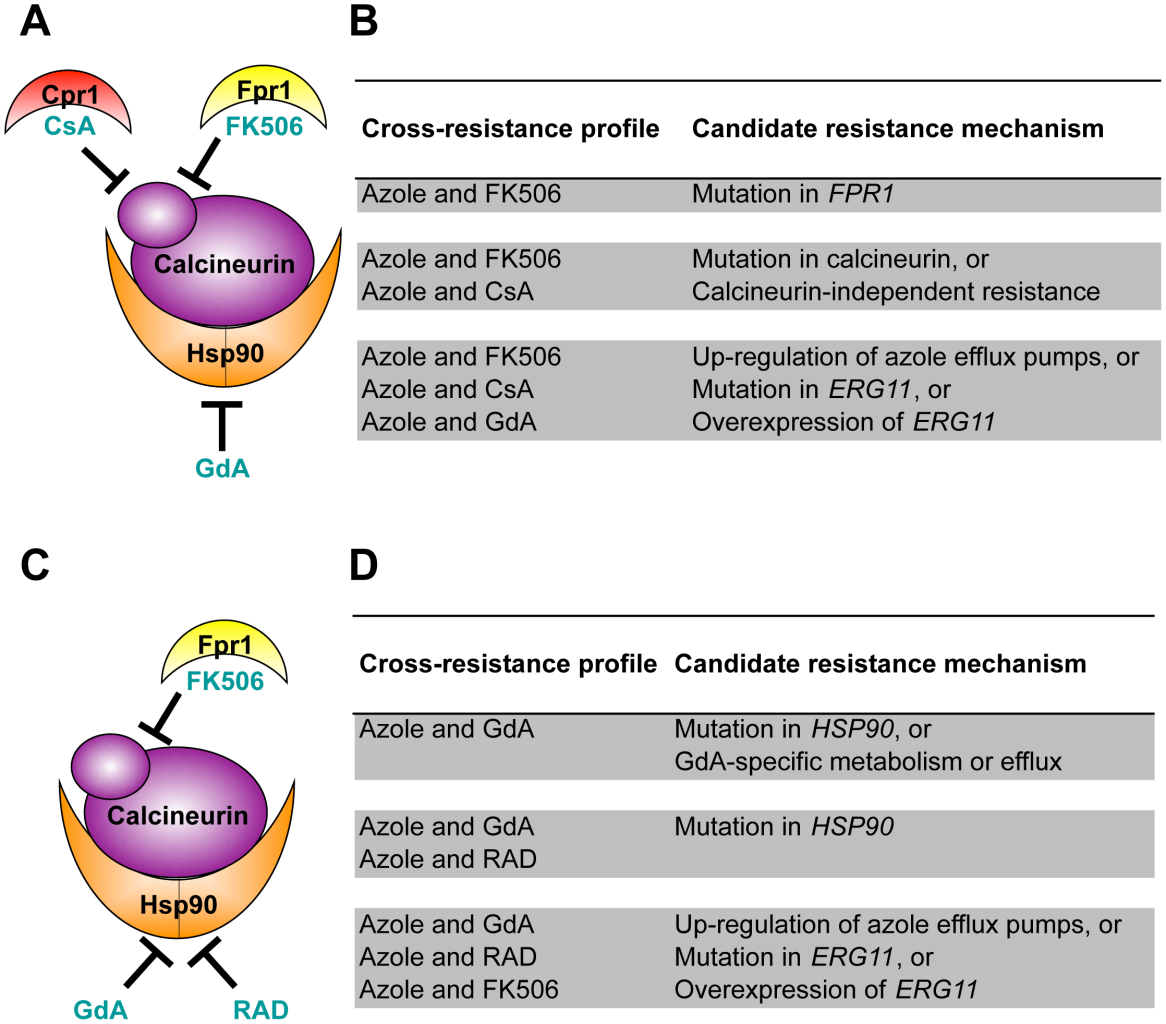
Cross-resistance profiles provide a strategy to predict resistance mechanisms. (A) Strains evolved in azole and FK506 were tested for cross-resistance to azole and the calcineurin inhibitor cyclosporin A as well as azole and the Hsp90 inhibitor geldanamycin. (B) Candidate resistance mechanisms based on specific cross-resistance profiles of strains evolved with azole and FK506. (C) Strains evolved in azole and geldanamycin were tested for cross-resistance to azole and the Hsp90 inhibitor radicicol as well as azole and the calcineurin inhibitor FK506. (D) Candidate resistance mechanisms based on specific cross-resistance profiles of strains evolved with azole and geldanamycin. GdA = geldanamycin; RAD = radicicol; and CsA = cyclosporin A.

To gain insight into mechanisms of resistance to the drug combinations, we assessed cross-resistance profiles. Cross-resistance assays were performed in the presence of a fixed concentration of an azole and a gradient of concentrations of the structurally dissimilar counterpart to the Hsp90 or calcineurin inhibitor with which the population was evolved (native inhibitor), as well as with an azole and an inhibitor of the other stress response regulator not targeted in the evolution experiment (naïve inhibitor; i.e. Hsp90 inhibitor if the population was evolved with a calcineurin inhibitor). Cross-resistance profiles can be used to predict candidate resistance mechanisms based on an understanding of how these inhibitors bind to and inhibit their targets (Figure 3).

Lineages evolved with azole and FK506 were assayed for resistance to azole and geldanamycin (a naïve inhibitor) as well as to azole and cyclosporin A (a structurally dissimilar calcineurin inhibitor) [39], [40] (Figure 3A). FK506 inhibits calcineurin by forming a complex with the immunophilin Fpr1, and it is the drug-immunophilin complex that binds to and inhibits calcineurin [41]. The structurally unrelated calcineurin inhibitor cyclosporin A binds to a distinct immunophilin, Cpr1, to form a complex that binds to calcineurin and inhibits its function [40]. Geldanamycin inhibits Hsp90 by binding directly to the unconventional Bergerat nucleotide-binding pocket of Hsp90 [42], [43]. The level of resistance to these specific drug combinations suggests several candidate mechanisms of resistance (Figure 3B). For example, resistance to the combination of azole and FK506 but not to azole and other inhibitors tested suggests an FK506-specific mechanism of resistance such as mutation of *FPR1*. If resistance was also observed to the combination of azole and cyclosporin A, this would suggest that calcineurin has been altered in a way that prevents the binding of both immunophilin-drug complexes, or that a calcineurin-independent mechanism of azole resistance has evolved. If resistance was also observed to the combination of an azole and the naïve inhibitor geldanamycin, this would suggest that resistance emerged by a mechanism that is independent of the stress response regulators Hsp90 and calcineurin; candidate mechanisms include those that block the effect of the azoles on their target, such as up-regulation of the drug efflux pump Pdr5 in *S. cerevisiae* [28], or alteration of the azole target Erg11 that prevents azole binding [29].

Lineages evolved with azole and geldanamycin were assayed for resistance to azole and radicicol, a structurally unrelated Hsp90 inhibitor. Like geldanamycin, radicicol binds to the unusual nucleotide-binding pocket of Hsp90, inhibiting its chaperone function [42] (Figure 3C). These lineages were also assayed for cross-resistance to azole and FK506, a naïve inhibitor to these strains. Resistance to azole and geldanamycin alone suggests that a mutation in *HSP90* occurred that prevents the binding of geldanamycin (Figure 3D). This cross-resistance profile is also consistent with a specific increase in geldanamycin metabolism or efflux. Cross-resistance to azole and FK506 suggests that an azole resistance mechanism evolved that is independent of the stress response regulators Hsp90 and calcineurin.

**Figure 4:**
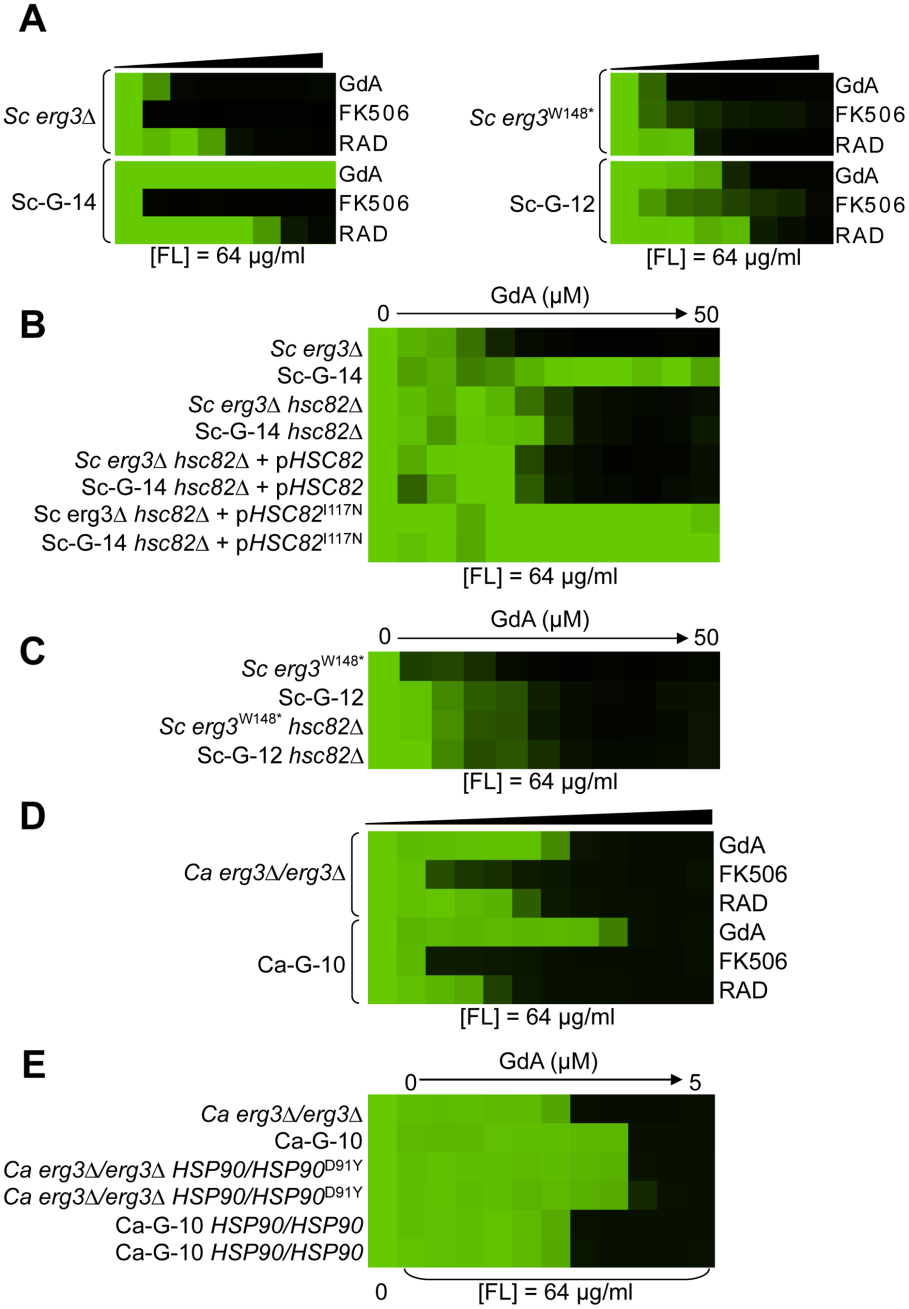
Mutations in *HSP90* confer resistance to azole and geldanamycin in two *S. cerevisiae* lineages and in one *C. albicans* lineage. (A) Sc-G-12 (right panel) and Sc-G-14 (left panel) are both resistant to azole and geldanamycin and slightly cross-resistant to azole and radicicol, relative to their parental strains (above). (B) Resistance to azole and geldanamycin in Sc-G-14 is attributable to *HSC82^I117N^*. Replacing the ancestral allele with the *HSC82^I117N^* allele expressed on a plasmid increases resistance of the ancestral strain to the level observed in Sc-G-14, while replacing the *HSC82^I117N^* allele with the ancestral allele on a plasmid abrogates resistance of Sc-G-14. (C) Deletion of *HSC82* in Sc-G-12 or its parental strain phenocopies resistance of Sc-G-12, suggesting that HSC82^K385*^ confers resistance by loss of function of *HSC82*. (D) Ca-G-10 has increased resistance to azole and geldanamycin but no cross-resistance to azole and FK506 or azole and radicicol. (E) Resistance to azole and geldanamycin in Ca-G-10 is attributable to *HSP90^D91Y^*. Replacing the native *HSP90* allele in parental strain with *HSP90^D91Y^* phenocopied resistance of Ca-G-10. Conversely, resistance of Ca-G-10 was abrogated when *HSP90^D91Y^* was replaced with the ancestral *HSP90* allele. Resistance assays were performed and analyzed is in Figure 2, after incubation at 30°C for 2 days (D) or 3 days (A–C, E). Assays were performed in YPD (A, C–E) or SD with amino acid supplements (B). GdA = geldanamycin; RAD = radicicol; and FL = fluconazole.

**Figure 5:**
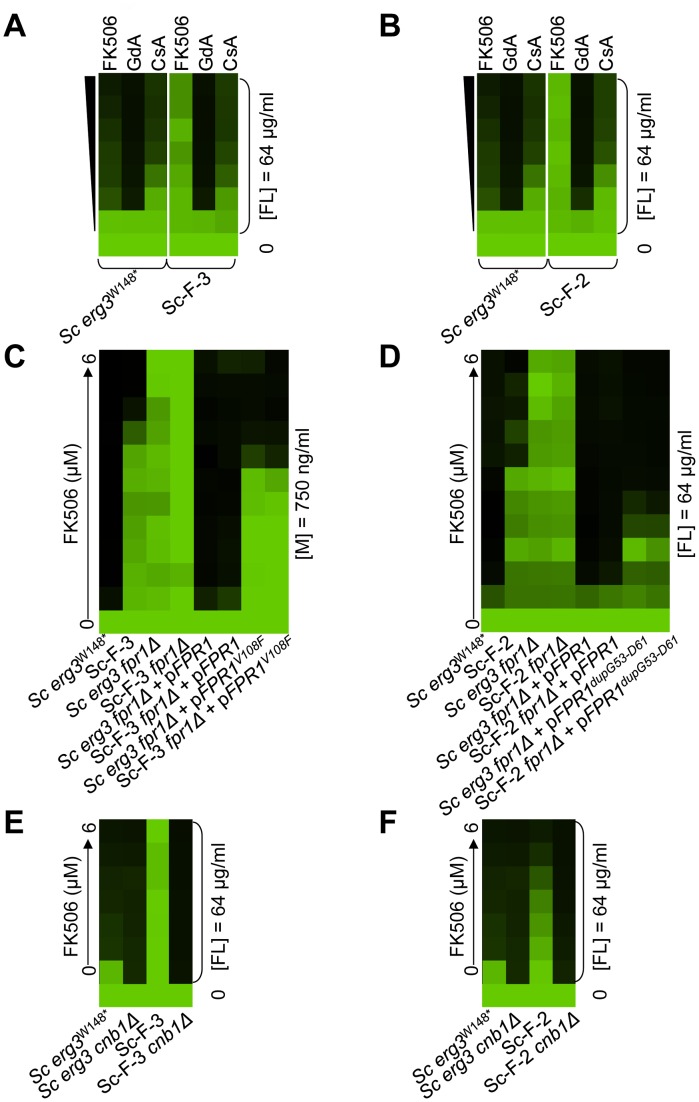
Mutations in *FPR1* confer resistance to azole and FK506 in two *S. cerevisiae* lineages. Sc-F-3 (A) and Sc-F-2 (B) evolved resistance to azole and FK506 but no cross-resistance to azole and either geldanamycin or cyclosporin A. (C) *FPR1^V108F^* confers resistance in Sc-F-3. Replacing the ancestral *FPR1* allele with *FPR1^V108F^* expressed from a plasmid increases resistance of the ancestral strain to a similar level as that observed in Sc-F-3. Conversely, replacing the *FPR1^V108F^* allele of Sc-F-3 with the ancestral *FPR1* allele expressed on a plasmid abrogates resistance of Sc-F-3. (D) *FPR1^dupG53-D61^* confers resistance in Sc-F-2. Replacing the ancestral *FPR1* allele with *FPR1^dupG53-D61^* expressed from a plasmid increases resistance of the ancestral strain, while replacing the *FPR1^dupG53-D61^* allele of Sc-F-2 with the ancestral *FPR1* allele expressed on a plasmid abrogates resistance of Sc-F-2. (E) The resistance phenotype of Sc-F-3 remains dependent on calcineurin. Deletion of the regulatory subunit of calcineurin necessary for its function, *CNB1*, abolished resistance to azole and FK506. (F) The resistance phenotype of Sc-F-2 remains dependent on calcineurin. Resistance assays were performed and analyzed is in Figure 2, with incubation for 2 days at 30°C in YPD (A, B, D, and F) or SD with amino acid supplements (C and E). CsA = cyclosporin A; GdA = geldanamycin; FL = fluconazole; and M = miconazole.

**Figure 6:**
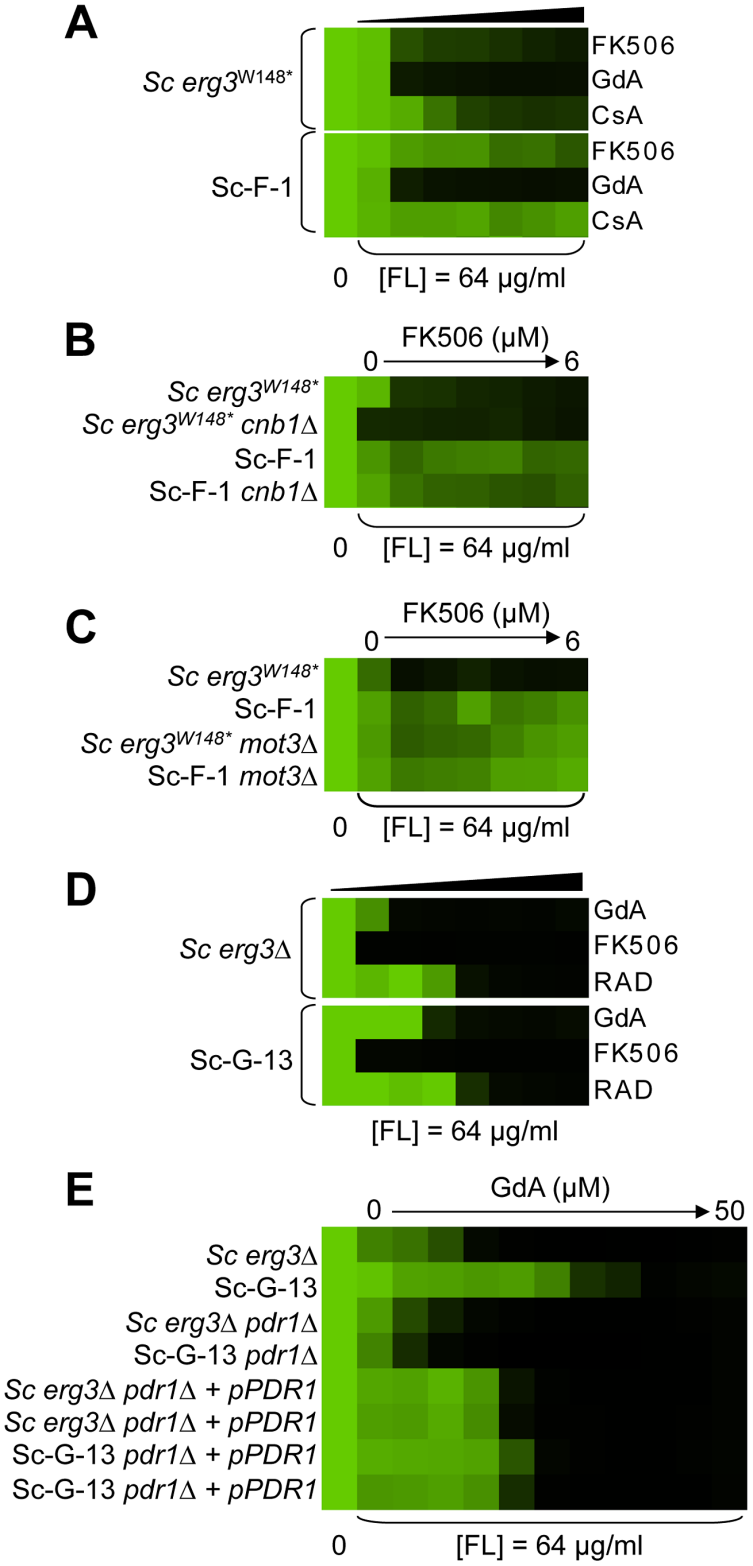
Whole-genome sequencing identifies mutations that confer resistance to azole and FK506, as well as azole and geldanamycin. (A) Sc-F-1 is resistant to azole and FK506 and cross-resistant to azole and cyclosporin A. (B) Resistance of Sc-F-1 is calcineurin-independent. Deletion of *CNB1*, which encodes the regulatory subunit of calcineurin required for its activation, does not affect resistance of Sc-F-1. (C) Deletion of *MOT3* in the ancestral strain confers resistance to azole and FK506 equivalent to Sc-F-1, which is consistent with the MOT3^G265*^ allele of Sc-F-1 conferring resistance to azole and FK506. (D) Sc-G-13 is slightly resistant to azole and geldanamycin. (E) Resistance to azole and geldanamycin in Sc-G-13 is reduced when *PDR^P865R^* is deleted and *PDR1* is expressed on a plasmid. Resistance assays were performed and analyzed as in Figure 2, with incubation for 2 days at 30°C in YPD (A–D) or SD (E). CsA = cyclosporin A; GdA = geldanamycin; RAD = radicicol; and FL = fluconazole.

**Figure 7:**
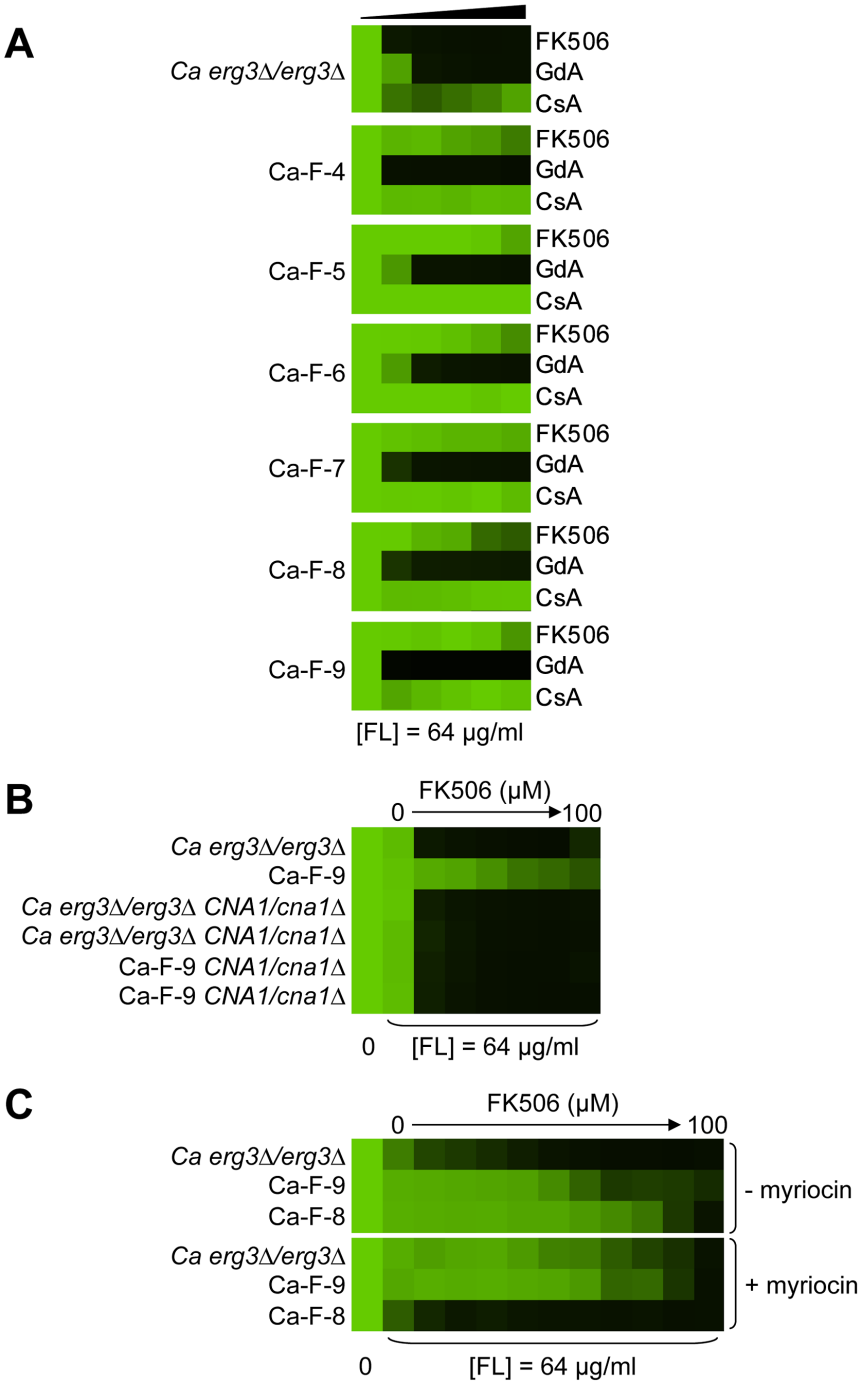
Six *C. albicans* lineages evolved with azole and FK506 share the same cross-resistance profile, and a mutation in *CNA1* and *LCB1* confers resistance. (A) Each *C. albicans* lineage is resistant to high concentrations of FK506 or cyclosporin A in the presence of azole. (B) *CNA1* ^S401*^ confers resistance to azole and calcineurin inhibitors in Ca-F-9. The C1201A mutation in *CNA1*, the gene encoding the catalytic subunit of calcineurin, leads to a premature stop codon and removal of the autoinhibitory domain. Deletion of *CNA1* ^S401*^ in Ca-F-9 abrogates resistance, while deletion of one allele of *CNA1* in the parental strain has no impact on sensitivity. (C) The A1169T mutation in orf19.6438 resulting in non-synonymous substitution (L390F) in this ortholog of *S. cerevisiae LCB1* likely confers resistance in Ca-F-8. Lcb1 encodes a component of serine palmitoyltransferase that is responsible for the first committed step in sphingolipid biosynthesis, along with Lcb2. Inhibition of Lcb1 and Lcb2 with myriocin (900 nM) abrogates resistance of Ca-F-8. Resistance assays were performed and analyzed is in Figure 2, with incubation for 2 days at 30°C in YPD. GdA = geldanamycin; CsA = cyclosporin A; and FL = fluconazole.

Variation in the patterns of cross-resistance to the distinct drug combinations was observed among the evolved strains (Figure 4, Figure 5, Figure 6, Figure 7), implicating a multitude of distinct resistance mechanisms. Even within a cross-resistance category variation was observed in the level of resistance to the drug combinations between strains, indicating that different mutations were responsible for resistance. This is consistent with the variation in levels of resistance with the native drug combination with which the population was originally evolved (Figure 2).

### Mutations in *HSP90* confer resistance to azole and geldanamycin in two *S. cerevisiae* lineages and one *C. albicans* lineage

Two *S. cerevisiae* lineages evolved with azole and geldanamycin, Sc-G-12 and Sc-G-14, displayed different levels of resistance to azole and geldanamycin relative to the ancestral strain (Figure 2C and 2D). Both Sc-G-12 and Sc-G-14 showed increased cross-resistance to azole and radicicol, although Sc-G-14 was able to grow with higher concentrations of azole and geldanamycin as well as azole and radicicol than Sc-G-12 (Figure 4A). Neither lineage showed any cross-resistance to azole and FK506. This suggests distinct mutations in *HSP90* might confer resistance to the drug combinations in these lineages. In *S. cerevisiae*, Hsp90 is encoded by two genes, *HSC82*, which is expressed at constitutively high levels, and *HSP82*, which is induced by high temperature [44]. Sequencing of *HSC82* and *HSP82* in Sc-G-14 identified a non-synonymous point mutation that maps to the N-terminal domain of *HSC82*, T1350A. This leads to the amino acid substitution I117N, a residue located in the groove lining the nucleotide-binding pocket of Hsp90 to which geldanamycin and radicicol bind. This residue is highly conserved and thought to be 90–100% buried [43]. The impact of *HSC82^I117N^* on resistance to azole and geldanamycin was confirmed by performing an allele swap, where *HSC82* was deleted from the ancestral strain and the evolved allele was introduced on a plasmid, and reciprocally, *HSC82* was deleted from the evolved strain and the ancestral allele was introduced. Expression of the *HSC82^I117N^* allele in the ancestral strain conferred a level of resistance to the combination of azole and geldanamycin equivalent to the evolved Sc-G-14 lineage (Figure 4B). Reciprocally, expression of only the ancestral *HSC82* allele in the evolved strain abrogated resistance to the drug combination (Figure 4B). This confirms that *HSC82^I117N^* confers resistance to the combination of azole and geldanamycin in the Sc-G-14 lineage, perhaps by blocking geldanamycin-mediated inhibition of Hsp90 function.

Sequencing of *HSC82* and *HSP82* in Sc-G-12 identified a 4 bp insertion in *HSC82* that results in a frameshift mutation and a premature stop codon in the middle of the coding sequence (HSC82^K385*^). This mutation is expected to render HSC82^K385*^ non-functional [45]. Surprisingly, deletion of *HSC82* in the parental strain confers a slight increase in resistance to azole and geldanamycin that phenocopies the resistance of Sc-G-12 (Figure 4C), suggesting that HSC82^K385*^ is indeed non-functional and confers resistance to the combination of azole and geldanamycin in Sc-G-12.

The *C. albicans* lineage Ca-G-10 exhibited increased resistance to azole and geldanamycin with no cross-resistance to azole and FK506 or azole and radicicol (Figure 4D). It was cross-resistant to azole and 17-AAG (Figure S1), a derivative of geldanamycin, suggesting a mode of resistance specific to ansamycin benzoquinone Hsp90 inhibitors. Sequencing identified a heterozygous, non-synonymous mutation in *HSP90*, G271T. This mutation causes a D91Y amino acid substitution at a residue in the Hsp90 nucleotide-binding pocket that is thought to be 60–90% buried. This residue is conserved in human Hsp90 although not in *S. cerevisiae*, where the native amino acid is glutamic acid [43]. To assess the impact of *HSP90^D91Y^* on resistance to the combination of azole and geldanamycin we performed an allele swap, replacing one allele of *HSP90* in the ancestral strain with the *HSP90^D91Y^* allele from the evolved strain, and replacing the *HSP90^D91Y^* allele in the evolved strain with the ancestral *HSP90* allele. Replacing *HSP90^D91Y^* in Ca-G-10 with the ancestral *HSP90* allele abrogated resistance in two independent transformants (Figure 4E). Reciprocally, replacing a native allele of *HSP90* in the ancestral strain with the *HSP90^D91Y^* allele conferred resistance that phenocopied that of Ca-G-10. This indicates that *HSP90^D91Y^* confers resistance to azole and geldanamycin and is responsible for resistance of Ca-G-10. Thus, distinct mutations in Hsp90 can block the impact of geldanamycin on azole resistance in both *C. albicans* and *S. cerevisiae*, providing a mechanism for resistance to this drug combination.

### Mutations in *FPR1* confer resistance to azole and FK506 in two *S. cerevisiae* lineages

Sc-F-2 and Sc-F-3 were evolved with azole and FK506, and demonstrate no cross-resistance to azole and cyclosporin A or azole and geldanamycin (Figure 5A and 5B), suggesting a mutation in *FPR1* may confer resistance to azole and FK506 in these lineages. Sequencing identified a non-synonymous mutation in Sc-F-3 *FPR1*, G322T. This mutation leads to a V108F amino acid substitution that was responsible for the azole and FK506 resistance, as determined by an allele swap where the *FPR1^V108F^* allele was expressed from a plasmid in the ancestral strain in which the native *FPR1* allele had been deleted, and reciprocally, the ancestral *FPR1* allele was expressed in Sc-F-3 in which the *FPR1^V108F^* allele had been deleted. Expression of the *FPR1^V108F^* allele in the ancestral strain conferred resistance to azole and FK506, while replacing *FPR1^V108F^* with the ancestral allele in Sc-F-3 abrogated resistance (Figure 5C). This mutation likely reduces but does not completely block binding of FK506 to Fpr1 given that complete deletion of *FPR1* confers an even greater level of resistance to azole and FK506. Consistent with this mutation conferring resistance to FK506 rather than altering the dependence of the azole resistance phenotype on calcineurin, deletion of the regulatory subunit of calcineurin required for its activation, *CNB1*, abrogated resistance of Sc-F-3 (Figure 5E).

Sequencing *FPR1* in Sc-F-2 revealed a tandem duplication of nine amino acids that maps to the middle of the coding sequence, *FPR^dupG53-D61^*. Expressing *FPR^dupG53-D61^* in the background of the ancestral strain conferred increased resistance to azole and FK506 (Figure 5D). That resistance was not as strong as in Sc-F-2 is likely due to the difference in expression levels of the native gene and the plasmid borne allele, which is driven by the *GPD1* promoter. It is unlikely that there are other mutations affecting resistance in Sc-F-2 given that the resistance phenotypes of the ancestral strain and Sc-F-2 with the plasmid borne *FPR^dupG53-D61^* allele as the sole source of Fpr1 were identical. Further confirming the importance of *FPR^dupG53-D61^* for resistance to azole and FK506, replacing *FPR^dupG53-D61^* of Sc-F-2 with the ancestral *FPR1* abrogated resistance (Figure 5D). As with the *FPR1* mutation identified in Sc-F-3, the *FPR^dupG53-D61^* mutation in Sc-F-2 likely reduces but does not block binding of FK506 to Fpr1 as deletion of *FPR1* confers an even greater level of resistance to azole and FK506 (Figure 5D). As with Sc-F-3, deletion of the regulatory subunit of calcineurin required for its activation, *CNB1*, abrogated resistance of Sc-F-2, consistent with this duplication in *FPR1* conferring resistance to FK506 rather than altering the dependence of the azole resistance phenotype on calcineurin (Figure 5F).

### Whole-genome sequencing identifies candidate resistance mutations in additional *S. cerevisiae* evolved lineages

Hypothesis driven approaches did not uncover any candidate resistance mutations for several evolved lineages. We therefore turned to whole genome sequencing to provide an unbiased approach to identify mutations that accompany the evolution of resistance to the drug combinations on a genomic scale. For example, *S. cerevisiae* Sc-F-1 was evolved with azole and FK506 and demonstrated robust resistance to the combination of azole and FK506 as well as azole and cyclosporin A (Figure 6A). This resistance profile suggested a possible mechanism of resistance involving alteration of calcineurin that prevents the binding of both protein-drug immunophilin complexes, or the emergence of a calcineurin-independent azole resistance mechanism. Calcineurin is encoded by the redundant catalytic subunits *CNA1* and *CNA2* and the regulatory subunit *CNB1* in *S. cerevisiae* [39], [46]. Sequencing of *CNA1, CNA2* and *CNB1* did not reveal any mutations. Intriguingly, abrogating calcineurin function by deletion of *CNB1* did not reduce resistance to azole and FK506 in Sc-F-1, indicating a calcineurin-independent mechanism of resistance had evolved (Figure 6B). Whole genome sequencing at high coverage (Table S1) identified two non-synonymous mutations (Table 2), as well as 58 mutations that were synonymous or in non-coding regions (Table S2 and Table S3); the best candidate for a mutation for affecting resistance was a mutation in *MOT3*, a transcriptional repressor of ergosterol biosynthesis genes [47]. The non-synonymous substitution in *MOT3* resulted in a premature stop codon near the middle of the coding sequence, MOT3^G265*^, suggesting that this might be a loss-of-function allele. Deletion of *MOT3* in the background of the ancestral strain or in Sc-F-1 phenocopied the level of resistance of Sc-F-1, which is consistent with MOT3^G265*^ being a loss-of-function allele that confers resistance in Sc-F-1 (Figure 6C).

**Table 2:** Non-synonymous *S. cerevisiae* single nucleotide variants.

Strain	Gene ID	Gene Name	GO Biological Process	Nucleotide Change	Non-synonymous Change
Sc-F-1	YMR070W	*MOT3*	cellular hyperosmotic response; negative regulation of ergosterol biosynthetic process; negative regulation of transcription from RNA polymerase II reporter; positive regulation of transcription from RNA polymerase II promoter	C792T	Q265*
Sc-F-1	YBL096C		unknown	T140G	N47K
Sc-G-13	YGL013C	*PDR1*	positive regulation of cellular response to drug; positive regulation of transcription from RNA polymerase II promoter	C2593G	P865R
Sc-G-13	YLR162W-A	*RRT15*	unknown	T81C	S28P
Sc-G-13	YGR090W	*UTP22*	maturation of SSU-rRNA from tricistronic rRNA transcript (SSU-rRNA, 5.8S rRNA, LSU-rRNA); rRNA processing; tRNA export from nucleus	G3648A	E1217K
Sc-G-13	YJR035W	*RAD26*	nucleotide-excision repair; transcription-coupled nucleotide-excision repair	G1590T	V531F
Sc-G-13	YFL017W-A	*SMX2*	mRNA splicing, via spliceosome	A148C	D50A

*S. cerevisiae* lineage Sc-G-13 was evolved with azole and geldanamycin and demonstrates only a small increase in resistance to this combination, with no cross-resistance to either azole and FK506 or azole and radicicol (Figure 6D). This resistance profile is consistent with a mutation in *HSC82* or *HSP82* that partially reduces binding of geldanamycin, however, no mutations were identified upon sequencing *HSC82* and *HSP82*. Genome sequencing of Sc-G-13 identified five non-synonymous mutations, as well as 130 that were synonymous or in non-coding regions (Table 2 and Table S3); the best candidate for a mutation affecting resistance was a C2593G mutation in *PDR1*, which encodes a transcription factor that regulates the expression of numerous multidrug transporters such as *PDR5*. Gain-of-function mutations in *PDR1* are a well-established mechanism of azole resistance that is independent of Hsp90 and calcineurin [16], [30], [48]. The mild resistance phenotype of Sc-G-13 suggested that the *PDR1^P865R^* allele in Sc-G-13 confers only a slight increase in drug efflux pump expression. Cross-resistance to azole and FK506 was not observed, likely because FK506 inhibits Pdr5-mediated efflux [49]. To evaluate the importance of the *PDR1^P865R^* allele in resistance to azole and geldanamycin we deleted *PDR1* from the ancestral strain and the evolved Sc-G-13 lineage and introduced the ancestral *PDR1* allele on a plasmid driven by the *GPD1* promoter. Replacing the *PDR1^P865R^* allele of Sc-G-13 with the ancestral *PDR1* allele reduced resistance of Sc-G-13 (Figure 6E). Resistance remained slightly increased relative to the ancestral strain, likely due to higher expression of *PDR1* from the *GPD1* promoter relative to the native promoter; consistent with this possibility, simply replacing the ancestral *PDR1* allele in the ancestor with the same allele on the plasmid conferred a small increase in resistance (Figure 6E). Since there was no difference in resistance phenotype between the ancestral and evolved strains when the plasmid provided the only allele of *PDR1*, there are likely no other mutations conferring resistance in Sc-G-13.

### Whole-genome sequencing identifies extensive aneuploidy in four of the *C. albicans* evolved lineages and additional candidate resistance mutations in two of the *C. albicans* lineages

For the six *C. albicans* lineages evolved with fluconazole and FK506 (Ca-F-4, Ca-F-5, Ca-F-6, Ca-F-7, Ca-F-8, and Ca-F-9), candidate resistance mutations were not identified by hypotheses-based cross-resistance profiles. These lineages shared the same cross-resistance profile of resistance to high concentrations of FK506 and increased resistance to cyclosporin A in the presence of azole (Figure 7A). This profile suggested that either a mutation in calcineurin preventing binding of both drug-immunophilin complexes occurred or a calcineurin-independent mechanism of resistance to azoles evolved. We sequenced the genome of all six lineages of this resistance class.

**Figure 8:**
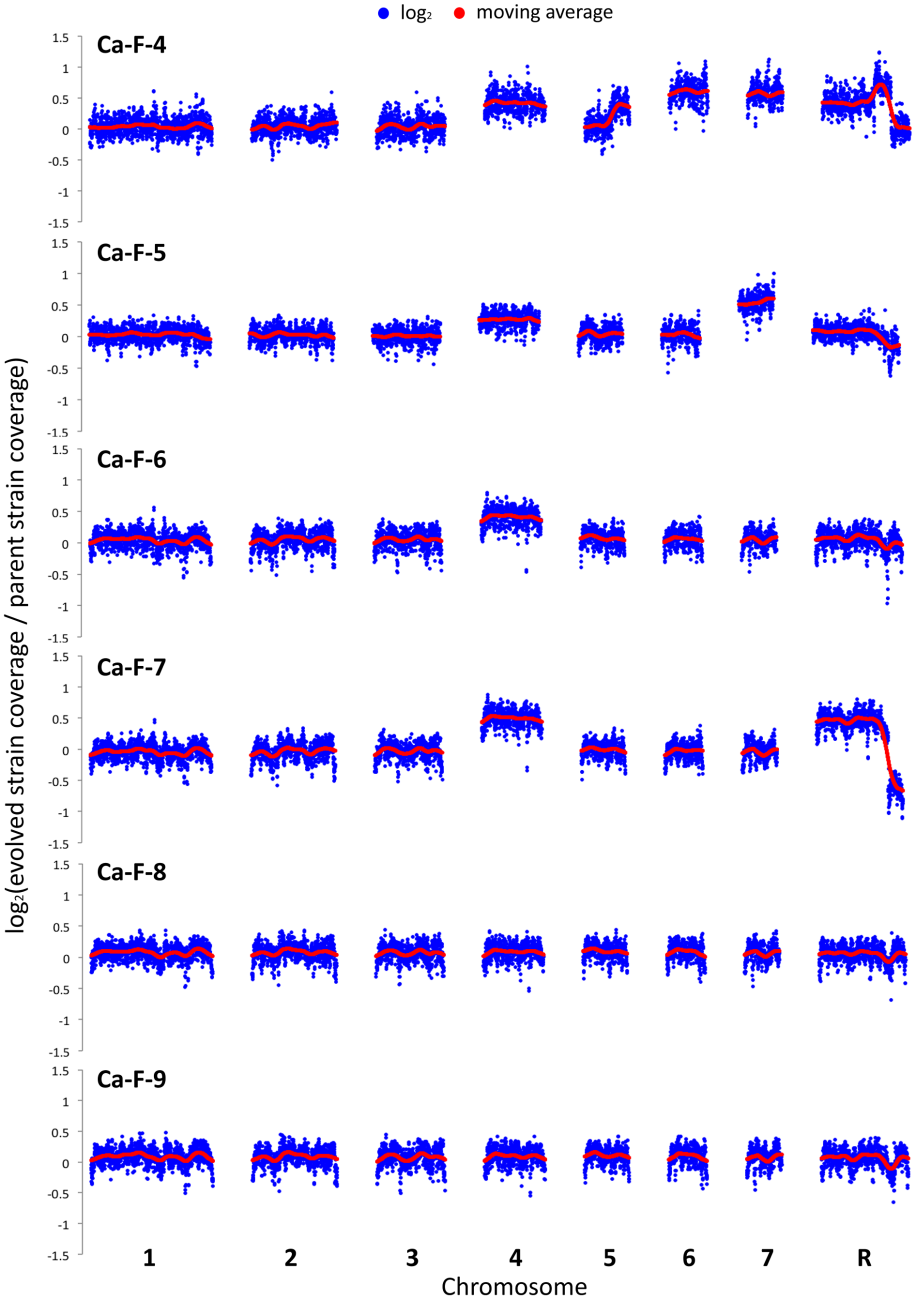
Aneuploidies identified in four *C. albicans* lineages that evolved resistance to the combination of azoles and calcineurin inhibitors. The genomes of six evolved strains were sequenced and profiled for copy number variants using CNV-Seq. Four of the strains contain aneuploidies: Ca-F-4, Ca-F-5, Ca-F-6, and Ca-F-7. Notably, chromosome 4 is increased in copy number in all four strains, suggesting that a locus on this chromosome is related to the mechanism of resistance. Blue = log_2_ values; Red = moving average values.

Genome analysis revealed aneuploidies in four of these evolved lineages. For Ca-F-4, we identified extensive aneuploidies in the absence of any non-synonymous mutations (Figure 8). This lineage exhibited increased copy number of chromosomes 4, 6 and 7 as well as an increase in copy number of the right arm of chromosome 5. Since approximately half the genome of Ca-F-4 had elevated copy number, resistance might be conferred by a combination of mechanisms including overexpression of the many relevant genes that were amplified including the gene encoding the drug transporter Mdr1, genes encoding ergosterol biosynthetic enzymes, the gene encoding the calcineurin regulatory subunit *CNB1*, or those encoding regulators of many other cellular pathways. We also identified increased copy number of chromosome 4 in three of the lineages, Ca-F-5, Ca-F-6 and Ca-F-7, as observed in Ca-F-4 (Figure 8). Ca-F-5 also had an increased copy number of chromosome 7. The remaining two lineages, Ca-F-8 and Ca-F-9, had no copy number variation other than variation in chromosome R, which was observed in all of the *C. albicans* lineages sequenced. Chromosome R contains the genes coding for rDNA, and extensive variation in size of the rDNA array has been observed in experimental populations of *C. albicans* [50], likely as a consequence of the highly repetitive nature of the genomic context. Two non-synonymous mutations were identified in *C. albicans* lineage Ca-F-9 (Table 3), and 7 mutations that were synonymous or in non-coding regions (Table S4). The best candidate for a resistance mutation is the C1201A mutation in *CNA1*, the gene encoding the catalytic subunit of calcineurin; this mutation leads to a premature stop codon, S401*. Truncation of *C. albicans* Cna1 at position 499 removes the autoinhibitory domain, resulting in a constitutively activated form of calcineurin [51]. Consistent with this mutation conferring resistance to the combination of azole and FK506 or azole and cyclosporin A, deletion of the evolved *CNA1* allele in Ca-F-9 abrogates resistance to the combination of azole and calcineurin inhibitor (Figure 7B). Deletion of one allele of *CNA1* in the ancestral strain has no effect on sensitivity to the drug combination. Thus, hyperactivation of calcineurin provides a mechanism by which resistance to azoles and calcineurin inhibitors can evolve.

**Table 3:** Non-synonymous *C. albicans* single nucleotide variants.

Strain	Gene ID	Gene Name	GO Biological Process	Nucleotide Change	Non-synonymous Change
Ca-F-6	orf19.3041		unknown	G376T	G126V
Ca-F-6	orf19.5015	*MYO2*	actin cytoskeleton reorganization; cell growth mode switching, budding to filamentous; cell morphogenesis; cellular response to heat; establishment of nucleus localization; establishment or maintenance of cell polarity; filamentous growth; filamentous growth of a population of unicellular organisms in response to heat; nucleus organization	G2404C	R802T
Ca-F-7	orf19.3041		unknown	G376T	G126V
Ca-F-8	orf19.2929	*GSC1*	(1,3)-β-D glucan biosynthetic process; fungal-type cell wall organization; pathogenesis	C1152A	H385N
Ca-F-8	orf19.3041		unknown	G376T	G126V
Ca-F-8	orf19.1263	*CFL1*	copper iron import; iron ion transport	G1591T	R531I
Ca-F-8	orf19.6131	*KSR1*	sphingolipid biosynthetic process	A21T	I8F
Ca-F-8	orf19.6438		predicted: sphingolipid biosynthetic process	A1169T	L390F
Ca-G-10	orf19.2174	*RAD57*	predicted: heteroduplex formation; meiotic DNA recombinase activity; reciprocal meiotic recombination; telomere maintenance via recombination	T341G	C114W
Ca-G-10	orf19.4337		monocarboxylic acid transport	C1627A	S543Y
Ca-G-10	orf19.6515	*HSP90*	cellular response to drug; cellular response to heat; filamentous growth; filamentous growth of a population of unicellular organisms; intracellular steroid hormone receptor signaling pathway; negative regulation of filamentous growth of a population of unicellular organisms; pathogenesis; protein folding; regulation of apoptotic process	G270T	D91Y
Ca-G-10	orf19.6693		predicted: proteolysis	A3306C	I1103L
Ca-F-9	orf19.6033	*CNA1*	cellular response to biotic stimulus; cellular response to cation stress; cellular response to starvation; filamentous growth; filamentous growth of a population of unicellular organisms; filamentous growth of a population of unicellular organisms in response to biotic stimulus; filamentous growth of a population of unicellular organisms in response to starvation; fungal-type cell wall organization; hyphal growth; pathogenesis; regulation of apoptotic process	C1201A	S401*
Ca-F-9	orf19.3041		unknown	G376T	G126V

Five non-synonymous mutations were identified in the *C. albicans* lineage Ca-F-8 (Table 3), and 16 mutations that were synonymous or in non-coding regions (Table S4). The best candidate for a resistance mutation is the A1169T mutation identified in orf19.6438 resulting in non-synonymous substitution, L390F. orf19.6438 remains uncharacterized in *C. albicans* but is an ortholog of *S. cerevisiae LCB1*, which encodes a component of serine palmitoyltransferase that is responsible for the first committed step in sphingolipid biosynthesis, along with Lcb2 [52]. Sphingolipids are a necessary component of the fungal cell membrane and have known interactions with ergosterol [53], while inhibitors of sphingolipid biosynthesis can enhance the efficacy of azoles [54]. To test the model that *LCB1* ^L390F^ confers resistance to the combination of azole and calcineurin inhibitor we used the serine palmitoyltransferase inhibitor myriocin, which inhibits Lcb1 and Lcb2 [55]. Inhibition of Lcb1 with myriocin abrogated resistance to azole and FK506 of the evolved lineage Ca-F-8 but did not affect resistance of Ca-F-9 (Figure 7C), suggesting that *LCB1* ^L390F^ confers resistance to the drug combination. Notably, myriocin caused an increase in resistance of the ancestral strain to azole and FK506 suggesting that resistance phenotypes are exquisitely sensitive to the balance of sphingolipid biosynthesis.

## Discussion

Our study provides the first experimental analysis of the evolution of resistance to drug combinations in fungi, illuminating the molecular basis of a transition of drug resistance from dependence on a key stress response regulator to independence, and a diversity of resistance mechanisms that can evolve in response to selection. This work addresses some of the most fundamental questions about the nature of adaptation. One key question is how many mutations underlie adaptive evolution. For all of the lineages for which we functionally tested the importance of mutations identified, we found that a single mutation was responsible for adaptation, in contrast to other experimental evolution studies with *S. cerevisiae* where multiple adaptive mutations were implicated [56], [57]. The small number of adaptive mutations identified in our study may reflect the short duration of the evolution experiment and the strength of the selection. Despite the limited number of adaptive mutations, we identified a larger number of total mutations in many lineages than reported in other studies [57]. The elevated number of mutations may be specific to the intense drug selection pressure, as bacterial mutation rates can increase in the presence of antibiotic selection [58], and antifungals have been associated with the rapid appearance of aneuploidies and genomic instability [59]. Another central question is how many genetic routes are there to adaptation. Among only 14 evolved lineages, we identified a diversity of adaptive mechanisms including target-based resistance to Hsp90 or calcineurin inhibitors and distinct mutations that render azole resistance independent of cellular stress response regulators, suggesting a complex adaptive landscape with multiple genotypes leading to high fitness adaptive peaks. Exploring the impact of the adaptive mutations on fitness in different environments, including in the absence of drug, will be key to understanding fitness costs of drug resistance, evolutionary trade-offs, and the limits of adaptation.

By starting the evolution experiment with strains that are resistant to azoles in a manner that depends on Hsp90 and calcineurin, we provide relevance for a clinical context where Hsp90 and calcineurin inhibitors could be deployed in combination with azoles to render azole-resistant isolates responsive to treatment. There is some precedent for the evolution of resistance to these drug combinations, as clinical isolates recovered from an HIV-infected patient over the course of two years evolved increased resistance to the combination of azole and inhibitors of Hsp90 or calcineurin [16]. While this patient was not treated with Hsp90 or calcineurin inhibitors, fever may have provided the selection for Hsp90 independence given that febrile temperatures cause global problems in protein folding that can overwhelm Hsp90 function and reduce azole resistance in a manner that phenocopies Hsp90 inhibition [16]. In our experimental evolution study, most of the 290 lineages initiated went extinct, while the 14 lineages that evolved resistance to the combination of azole and inhibitor of Hsp90 or calcineurin acquired a diversity of resistance mechanisms. These resistance mechanisms included mutations that rendered *erg3*-mediated azole resistance independent of the stress response regulator calcineurin, mutations that blocked the effects of the Hsp90 or calcineurin inhibitor, and large-scale aneuploidies. This experimental evolution approach provides a powerful system to predict the mechanisms by which resistance to drug combinations may evolve in the clinic. Consistent with the relevance of our findings, the increased resistance to azole and inhibitor of Hsp90 or calcineurin in isolates that evolved in an HIV-infected patient was accompanied by mutations causing overexpression of multidrug transporters [16], [60], as expected for the *PDR1* mutation identified in one of our lineages.

One of the most prevalent mechanisms of resistance identified in our evolved populations was mutation in the target of the drug used in combination with azole during the evolution experiment. For Hsp90 inhibitors, it has been predicted that the probability of target-based resistance would be relatively low given that the amino acid residues in the nucleotide-binding site of Hsp90 family members are highly conserved from bacteria to mammals [61], suggesting that mutations that confer resistance would likely inactivate this essential molecular chaperone. This has helped fuel research on Hsp90 as a target for development of anti-cancer drugs, where inhibiting Hsp90 can impair the function of a multitude of oncoproteins [62]–[64]. Despite the constraints, there is precedent for point mutations in Hsp90 conferring resistance to Hsp90 inhibitors. One study engineered *S. cerevisiae* strains to be hypersensitive to drugs and expressed yeast or human Hsp90 as the sole source of the chaperone; introduction of a single point mutation (A107N for yeast, A121N for human Hsp90α, and A116N for human Hsp90β) conferred resistance to Hsp90 inhibitors [65]. Further, the fungus that produces radicicol, *Humicola fuscoatra*, harbours an Hsp90 with reduced binding affinity to radicicol but not geldanamycin [66]. Three of our evolved lineages acquired substitutions in Hsp90 that rendered *erg3*-mediated azole resistance more recalcitrant to the effects of Hsp90 inhibitors (Figure 4). For one *S. cerevisiae* lineage (Sc-G-14) and one *C. albicans* lineage (Ca-G-10), the mutations were in the nucleotide-binding domain, consistent with impairing drug binding. For *S. cerevisiae* lineage Sc-G-12, the mutation led to a premature stop codon (K385*); consistent with this *HSC82* mutation causing a loss of function, deletion of *HSC82* in the parental strain phenocopied resistance of Sc-G-12. Reducing dosage of a drug target often confers hypersensitivity to the drug rather than resistance [67]; this may suggest compensatory upregulation of the other *S. cerevisiae* gene encoding Hsp90, *HSP82*, which could confer elevated resistance. Target-based resistance to Hsp90 inhibitors has yet to emerge in Hsp90 inhibitor clinical trials, suggesting that these mutations may be associated with a fitness cost.

Mutations in the drug target also emerged as a mechanism that renders *erg3*-mediated azole resistance recalcitrant to the effects of calcineurin inhibitors in our evolved lineages. Two *S. cerevisiae* lineages acquired mutations in *FPR1*, which encodes the immunophilin that FK506 must bind to in order to form the protein-drug complex that inhibits calcineurin function [41]. A V108F substitution was identified in Sc-F-3 and a nine amino acid duplication near the protein midpoint was identified in Sc-F-2 (dupG53-D61). These alterations likely reduce but do not block FK506 binding, given that deletion of *FPR1* conferred a higher level of FK506 resistance (Figure 5). There is precedent for overexpression or disruption of *FPR1* conferring resistance to FK506 in *S. cerevisiae* [68], as well as for a W430C amino acid substitution in one of the two redundant calcineurin catalytic subunits Cna2 [69]. One *C. albicans* lineage, Ca-F-9, acquired a mutation in the catalytic subunit of calcineurin, *CNA1^C1201A^*, which results in a S401* premature stop codon that confers resistance to azole and both FK506 and cyclosporin A (Figure 7), likely due to hyperactivation of calcineurin [51]. Despite the emergence of target-based resistance to calcineurin inhibitors *in vitro*, there may be significant constraints that minimize the emergence of resistance in the human host. FK506 (tacrolimus) and cyclosporin A are front line immunosuppressants broadly used in the clinic to inhibit calcineurin function, thereby blocking T-cell activation in response to antigen presentation and suppressing immune responses that contribute to transplant rejection [39], [70]. Invasive fungal infections occur in ∼40% of transplant recipients including those that receive a calcineurin inhibitor as an immunosuppressant [71], however, this immunosuppressive therapy does not select for resistance to calcineurin inhibitors in *C. albicans* or *Cryptococcus neoformans* recovered from these patients [72], [73]. That resistance has not been observed in the host suggests that the resistant mutants may have reduced fitness relative to their sensitive counterparts or that other selective constraints alter the evolutionary dynamics.

Several of our evolved lineages took a distinct evolutionary trajectory, and evolved azole resistance mechanisms that are independent of the cellular stress response regulators. *S. cerevisiae* lineage Sc-F-1 evolved cross-resistance to azole and FK506 as well as azole and cyclosporin A (Figure 6). The azole resistance phenotype was independent of calcineurin but dependent on Hsp90 (Figure 6), suggesting a resistance mechanism that is contingent upon distinct Hsp90 downstream effectors, such as Mkc1 [74]. We identified an adaptive mutation in *MOT3* (Table 2), a transcriptional repressor of ergosterol biosynthesis genes [47], which resulted in a premature stop codon, G265* and likely a loss-of-function allele (Figure 6C). Loss of function of Mot3 would lead to overexpression of ergosterol biosynthesis genes, which could minimize the impact of azoles on their target or could lead to a change in sterol balance that reduces the dependence of azole resistance on calcineurin. Changes in membrane composition may also explain the resistance of *C. albicans* lineage Ca-F-8 to azoles and calcineurin inhibitors, which was attributed to a mutation in the ortholog of *S. cerevisiae LCB1* (Figure 7), encoding a regulator of sphingolipid biosynthesis. Notably, Mot3 is also a prion protein, which can convert between structurally and functionally distinct states, at least one of which is transmissible [75]; changes on Mot3 conformation and activity can modulate phenotypic variation in *S. cerevisiae*, and thus may influence the evolution of drug resistance phenotypes. *S. cerevisiae* lineage Sc-G-13 evolved a small increase in resistance to azole and geldanamycin associated with a mutation in *PDR1*, which encodes a transcription factor that regulates the expression of drug transporters such as *PDR5* (Figure 6). Gain-of-function mutations in *PDR1* are known to confer azole resistance that is independent of Hsp90 and calcineurin [16], [30], [48]. Cross-resistance to azole and FK506 may not have been observed because FK506 inhibits Pdr5-mediated efflux [49]. The weak resistance phenotype could reflect a small increase in transporter expression, or a fitness cost of the *PDR1* mutation in an *erg3* mutant background [76].

Several of the *C. albicans* lineages that evolved resistance to azole and calcineurin inhibitors demonstrated a complex genomic landscape of aneuploidies. The emergence of azole resistance in *C. albicans* has been associated with general aneuploidies as well as the formation of a specific isochromosome composed of two left arms of chromosome 5 (i5L) [77]. The isochromosome confers azole resistance due to increased dosage of two genes located on the left arm of chromosome 5: *ERG11*, which encodes the target of the azoles; and *TAC1*, which encodes a transcriptional regulator of multidrug efflux pumps [78]. Our lineages were resistant to azoles at the outset of the experiment, suggesting that the aneuploidies emerged in response to stress or were selected as a mechanism of resistance to the drug combination. Ca-F-4, Ca-F-5, Ca-F-6, and Ca-F-7 all had numerous aneuploidies relative to the parental strain (Figure 8). One aneuploidy that was common to all four lineages was increased copy number of chromosome 4, suggesting that an important resistance determinant might reside on this chromosome. While one might predict that such aneuploidies would be associated with a fitness cost, it is notable that a previous analysis of isolates carrying the i5L isochromosome demonstrated improved fitness in the presence and absence of azoles, relative to their drug-sensitive counterpart [59]. In contrast, many azole resistance mutations are associated with a fitness cost [79], though this cost can be mitigated with further evolution [80]. The prevalence of aneuploidies in the *C. albicans* lineages underscores the remarkable genomic plasticity of this pathogen [81], and the diversity of genomic alterations that can accompany adaptation.

The landscape of genetic and genomic changes observed in our evolved lineages illuminate possible mechanisms by which resistance to drug combinations might evolve in the human host and suggest candidate targets to minimize the emergence of resistance. Despite optimizing our selection conditions to favour the evolution of resistance to the drug combination, the majority of lineages went extinct (Figure 1). Consistent with constraints that minimize the evolution of resistance to these drug combinations, treatment of organ transplant patients with calcineurin inhibitors has not yielded resistance to these drugs in fungal pathogens recovered from these patients despite the extensive use of these drugs in patient populations [72], [73]. While Hsp90 inhibitors remain at the clinical trial stage for cancer and other diseases [62], [63], [82], [83], resistance has yet to emerge in these patient populations. Although there are a multitude of mechanisms that can confer resistance to the drug combinations, they may not be favoured due to fitness costs in the complex host environments.

The mechanisms by which resistance to the drug combinations evolved in our lineages suggest novel targets that could be exploited to block the evolution of drug resistance. Drug interactions have tremendous potential to influence the evolution of drug resistance [84]. Elegant studies with antibacterials emphasize that the impact of these interactions are often more complex than anticipated [8], [85]–[87]. While synergistic interactions that yield inhibitory effects larger than expected from individual drugs can maximize the rate at which infection is cleared, antagonistic interactions that yield inhibitory effects smaller than expected can suppress the evolution of multi-drug resistance. Ultimately, a systems biology approach incorporating experimental evolution, genetics and genomics, and clinical samples will be crucial for the development of effective strategies to enhance the efficacy of antimicrobial agents and minimize the evolution of drug resistance.

## Materials and Methods

### Strain construction and culture conditions

All *Saccharomyces cerevisiae* and *Candida albicans* strains were archived in 25% glycerol and maintained at −80°C. Strains were typically grown and maintained in rich medium (YPD: 1% yeast extract, 2% bactopeptone, 2% glucose, with 2% agar for solid medium only), or in synthetic defined medium (SD, 0.67% yeast nitrogen base, 2% glucose, with 2% agar for solid medium only), supplemented with amino acids, as indicated. Strains were transformed using standard protocols. Strains used in this study are listed in Table S5. Strains were constructed as described in Text S1.

### Plasmid construction

Plasmids were constructed using standard recombinant DNA techniques. Plasmids used in this study are listed in Table S6 and oligonucleotides used in this study are listed in Table S7. Plasmids were constructed as described in Text S1. All plasmids were sequenced to confirm the absence of spurious non-synonymous mutations.

### Evolution experiment

Evolution experiments were initiated with three ancestral strains of *erg3*-mediated azole resistant strains: two haploid *S. cerevisiae* strains (*erg3Δ* and *erg3* ^W148*^) and one *C. albicans* strain (*erg3Δ*/*erg3Δ*; see Table S5). A founder colony was established for each ancestral strain and grown overnight in liquid, rich medium (YPD) without drug. From here, culture was transferred to a plate containing YPD with combinatorial drug concentrations of azole (fluconazole or miconazole) and geldanamycin, or azole (fluconazole or miconazole) and FK506 (i.e. treatments; see Table 1). Geldanamycin and FK506 were selected based on their specificity of target inhibition and their capacity to abrogate *erg3*-mediated azole resistance [16]; fluconazole and miconazole were selected as clinically relevant azoles of the triazole and imidazole class, respectively [4], [37]. Treatments were selected for the evolution experiment based on growth phenotype in the dose response matrices (Figure S2), such that strong directional selection for resistance would be applied. Concentrations were also varied to favour the emergence of distinct mechanisms of resistance. Lineages were then propagated in replicate in either 96-well plates (Sarstedt; 48 lineages initiated in this format) or 24-well plates (Becton Dickinson Labware; 242 lineages initiated in this format). The plates were formatted as described in Figure 1B. For propagation in 96-well plates, 1 µl of culture was transferred from the overnight culture to a final volume of 100 µl. Lineages were grown in a Tecan GENios plate reader and incubator at 30°C with constant agitation for three days. Subsequently, 1 µl of culture was transferred to a new plate containing YPD and treatment. Transfers occurred every 3 days to allow slow growing lineages to reach carrying capacity. This process was repeated until robust growth was present in some treatment wells. The experimental design for lineages propagated in 24-well plates was the same with the following adjustments: different drug combinations were selected for treatments; 10 µl of culture was transferred to 990 µl of YPD with treatment; plates were maintained at 30°C with constant agitation in a shaking incubator and transfers occurred every two days. With this dilution factor of 1/100, ∼6.6 generations occurs between transfers. The effective population size per lineage of ∼4.6×10^6^ was estimated as described [88], given that cultures reached saturation of ∼10^7^ cells/ml between transfers. Lineages that demonstrated reproducible resistance to the drug combination in which they were propagated were archived. Lineages unable to grow in the presence of the drug combination, either from when the cultures were initiated or over the course of the evolution experiment, were considered extinct. A summary of treatment concentrations, number of transfers and type of plate evolved in can been found in Table 1.

### Minimum inhibitory concentration and checkerboard assays

Resistance to drug combinations was assayed in 96-well microtiter plates, as previously described [16], [21]. Minimum inhibitory concentration (MIC) assays were set up to a final volume of 0.2 ml/well. MICs were performed in the absence of fluconazole (Sequoia Research Products) or with a constant concentration of fluconazole or miconazole (Sigma–Aldrich Co.), as indicated in the figures. All gradients were two-fold dilutions per step, with the final well containing no drug. The starting concentration of geldanamycin (Invivogen) gradients was 50 µM for *S. cerevisiae* strains and 5 µM for *C. albicans* strains. The starting concentration of FK506 (A.G. Scientific) gradients was 6 µM for *S. cerevisiae* strains and 100 µM for *C. albicans* strains. The starting concentration of radicicol (A.G. Scientific) gradients was 25 µM for both *S. cerevisiae* and *C. albicans* strains. The starting concentration of cyclosporin A (Calbiochem) gradients was 50 µM for both *S. cerevisiae* and *C. albicans* strains. The cell densities of overnight cultures were determined and diluted to an inoculation concentration of ∼10^3^ cells/well. Plates were incubated at 30°C in the dark for the period of time specified in the figure legend. Cultures were resuspended and absorbance at 600 nm was determined using a spectrophotometer (Molecular Devices) and corrected for background of the corresponding medium. OD measurements were standardized to either drug-free or azole-only control wells, as indicated. Data was plotted quantitatively with colour using Java Treeview 1.1.3 (http://jtreeview.sourceforge.net/). Resistance phenotypes were assessed on multiple occasions and in duplicate on each occasion with concordant results, validating that the phenotypes are reproducible and stable.

Dose response matrices, or checkerboard assays, were performed to a final volume 0.2 ml/well in 96-well microtiter plates, as previously described [74]. Two-fold dilutions of fluconazole were titrated along the X-axis from a starting concentration of 256 µg/ml, with the final row containing no fluconazole. Along the Y-axis, either geldanamycin or FK506 was titrated in two-fold dilutions with the final column containing no geldanamycin or FK506. The starting concentration of geldanamycin was 5 µM for checkerboards with either *S. cerevisiae* or *C. albicans* strains. The starting concentration of FK506 was 4 µM for checkerboards with *S. cerevisiae* and 40 µM for checkerboards with *C. albicans* strains. Concentrations were selected to cover a range that spanned from no effect on growth to near complete inhibition of growth. Plates were inoculated and growth assessed as was performed for MIC assays.

Fluconazole was dissolved in sterile ddH_2_O. The Hsp90 inhibitors geldanamycin and radicicol and the calcineurin inhibitors FK506 and cyclosporin A were dissolved in DMSO. Myriocin (Sigma) was dissolved in methanol.

### Genome sequencing

*C. albicans* cell pellets were digested with R-Zymolase for 1 hour (Zymo Research, D2002), prior to genomic DNA extraction with phenol-chloroform (EMD Millipore, EMD6810), and sodium acetate precipitation. Whole genome libraries were prepared using Nextera XT kits (Illumina, FC-131-1096) according to manufacturer's protocol. Libraries were sequenced on the Illumina HiSeq2000 platform using paired reads (101 bp) and version 3 reagents and chemistry.

The yeast genomes were sequenced in a multiplexed format, where an oligonucleotide index barcode was embedded within adapter sequences that were ligated to genomic DNA fragments [89]. Only one mismatch per barcode was permitted to prevent contamination across samples. Next, the sequence reads were filtered for low quality base calls trimming all bases from 5′ and 3′ read ends with Phred scores < Q30. Trimming sequence reads for low quality base calls drastically lowered false positive SNV calls.

De-multiplexed and trimmed reads from the *S. cerevisiae* strains were aligned to the *S288c 2010* genome, a high fidelity sequence from an individual yeast colony (from F. Dietrich's lab at Duke University; it is the SGD reference genome as of February 2011) [90]. Reads from the *C. albicans* strains were aligned to the SC5314 genome from CGD [91]. While *C. albicans* is an obligate diploid, the current build of the genome, assembly 21, is a haploid genome, and is more accurate than the original diploid genome, assembly 19 [92], [93]. The diploid assembly was not used because it features 412 supercontigs with non-obvious heterozygosity, whereas the haploid assembly has been curated and organized into 8 chromosomes [93].

Sequence reads were aligned with Bowtie2, which was chosen over other commonly used short-read aligners such as Illumina's Eland, Maq, SOAP and BWA because it has been reported to be one of the fastest accurate aligners [94]–[98]. Additionally, it was chosen because it is updated frequently, supports variable read lengths within a single input file, is multi-threaded with a minimal memory and temporary file footprint and supports the standard Sequence Alignment/Map (SAM) file format [94], [98]–[100]. Alignments and all subsequent sequence data were visualized using the Savant Genome Browser [101]. The average coverage of each genome was calculated and was sufficient for confident variant detection (Table S1).

Aligned sequence reads for *S. cerevisiae* were subsequently processed using the UnifiedGenotyper package of the Genome Analysis Toolkit (GATK), which features a comprehensive framework for discovering SNVs and calculating coverage across genomic data [102], [103]. Variants detected in the *S. cerevisiae* parental strains were subtracted from complete variant lists, yielding a set of novel variants that emerged during strain growth in the presence of drug. Since *C. albicans* is diploid, we processed the reads with a more accurate approach using the probabilistic framework JointSNVMix, which uses paired parental and evolved strain sequence data to determine significant novel variants [104]. After identifying candidate SNVs, the threshold for homozygous SNV calls for both haploid (*S. cerevisiae*) and diploid (*C. albicans*) systems was set to 85% alternate (non-reference) basecalls at a specific position. In a diploid system, 35% was the threshold set to identify heterozygous SNVs. All variant positions required a minimum coverage of 15× to be considered as a candidate SNV. The total number of high-confidence novel mutations agrees with mutation rates observed previously for *S. cerevisiae* (Table S3) [105]. To further verify that the sequence data are of high quality, we compared two distinct sequence runs from two different sequence library preparations of the same parent *C. albicans* strain CaLC660. The total number of diploid single nucleotide variants that exist between the parent strain and the reference genome (SC5314) is 3748, therefore there is 99.99% concordance between both sequence replicates (Table S8).

The software package CNV-seq was used to identify chromosomal regions that varied in copy number between parental strains and evolved lineages [106]. This analysis found no significant CNVs in the *S. cerevisiae* strains, but numerous large variants were observed in *C. albicans*.

Sequence data is publicly available on the NCBI Short Read Archive with accession SRA065341.

## Supporting Information

Figure S1 Ca-G-10 is slightly resistant to azole and geldanamycin, and slightly cross-resistant to azole and 17-AAG, a structural derivative of geldanamycin. Resistance assays were performed in YPD, with incubation for 2 days at 30°C. Optical densities were averaged for duplicate measurements and normalized relative to drug-free controls (see colour bar). GdA = geldanamycin; and M = miconazole.
(TIF)

Figure S2 Dose response matrices were used to select concentrations for the evolution experiment. Dose response matrices for parental strains: (A, B) *Sc erg3* ^W148*^; (C, D) *Sc erg3Δ*; and (E, F) *Ca erg3Δ/erg3Δ*. Monitoring growth in the concentration gradients of azole and geldanamycin (A, C, and E) or azole and FK506 (B, D, and F) was used to determine the concentrations of the drug combinations for the experimental evolution study in order to reduce growth relative to a no-drug control, but minimize the probability of extinction. Evolution experiments were initiated with the drug combinations indicated by the orange circles (24-well plates) and purple circles (96-well plates). Resistance assays were performed as in Figure S1, with incubation for 3 days at 30°C. FL = fluconazole; and GdA = geldanamycin.
(TIF)

Table S1 Mean coverage for whole genome sequenced strains.
(DOCX)

Table S2 Number of high confidence single nucleotide variants (coding and non-coding).
(DOCX)

Table S3 High confidence single nucleotide variants (coding and non-coding) identified in *S. cerevisiae*.
(XLSX)

Table S4 High confidence single nucleotide variants (coding and non-coding) identified in *C. albicans*.
(XLSX)

Table S5 Strains used in this study.
(DOCX)

Table S6 Plasmids used in this study.
(DOCX)

Table S7 Oligonucleotides used in this study.
(DOCX)

Table S8 Comparison of two sequence runs of the ancestral *C. albicans* strain CaLC660.
(XLSX)

Text S1 Supporting Materials and Methods.
(DOC)
