# Supplementary figures and images for: Genetic and Genomic Architecture of the Evolution of Resistance to Antifungal Drug Combinations
Source: PLoS Genet. 2013 Apr 4;9(4):e1003390. doi: 10.1371/journal.pgen.1003390 (PMC3617151; doi:10.1371/journal.pgen.1003390)

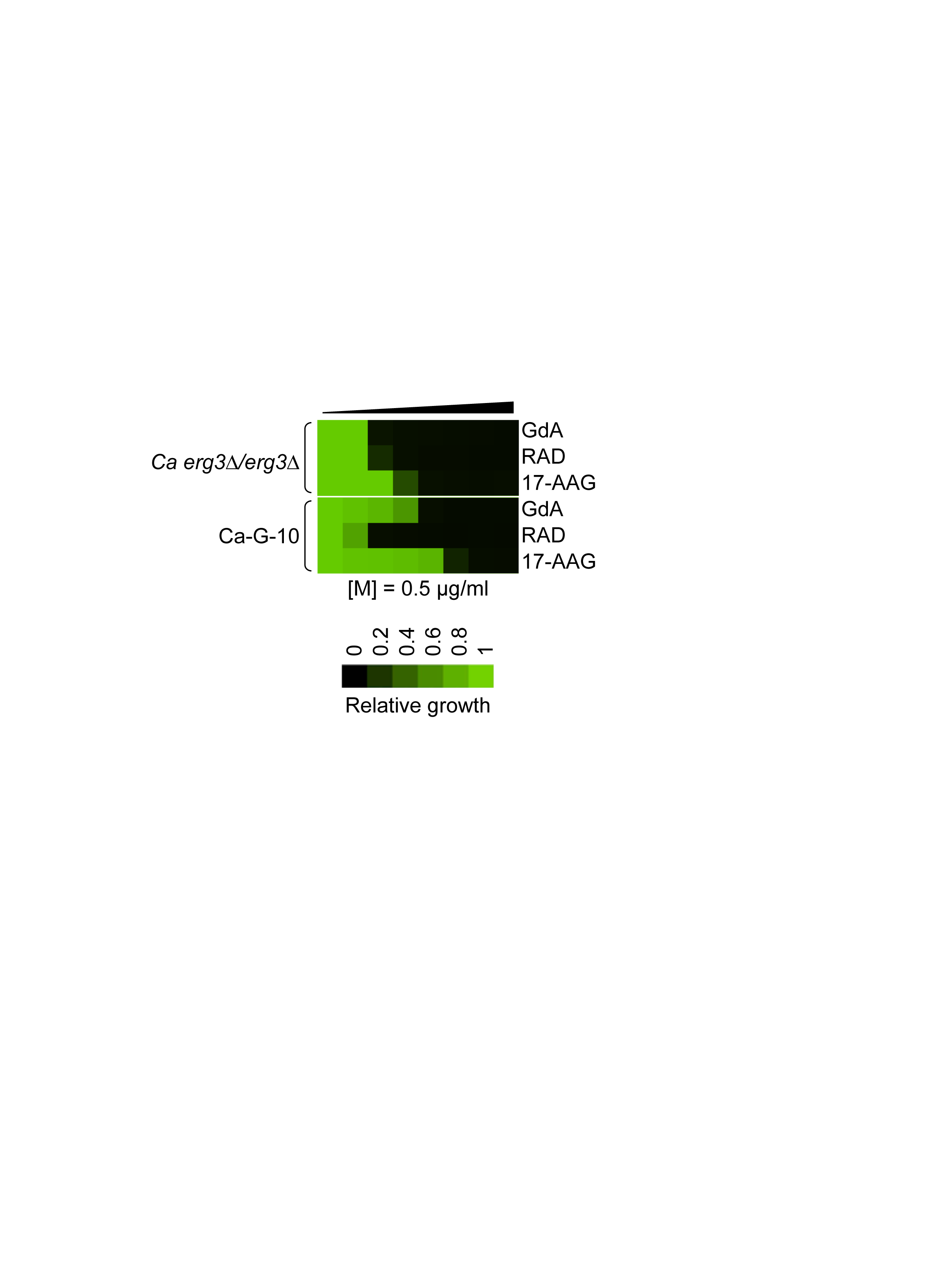

Supplement: Figure S1 — Ca-G-10 is slightly resistant to azole and geldanamycin, and slightly cross-resistant to azole and 17-AAG, a structural derivative of geldanamycin. Resistance assays were performed in YPD, with incubation for 2 days at 30°C. Optical densities were averaged for duplicate measurements and normalized relative to drug-free controls (see colour bar). GdA = geldanamycin; and M = miconazole. (TIF) [file pgen.1003390.s001.tif]

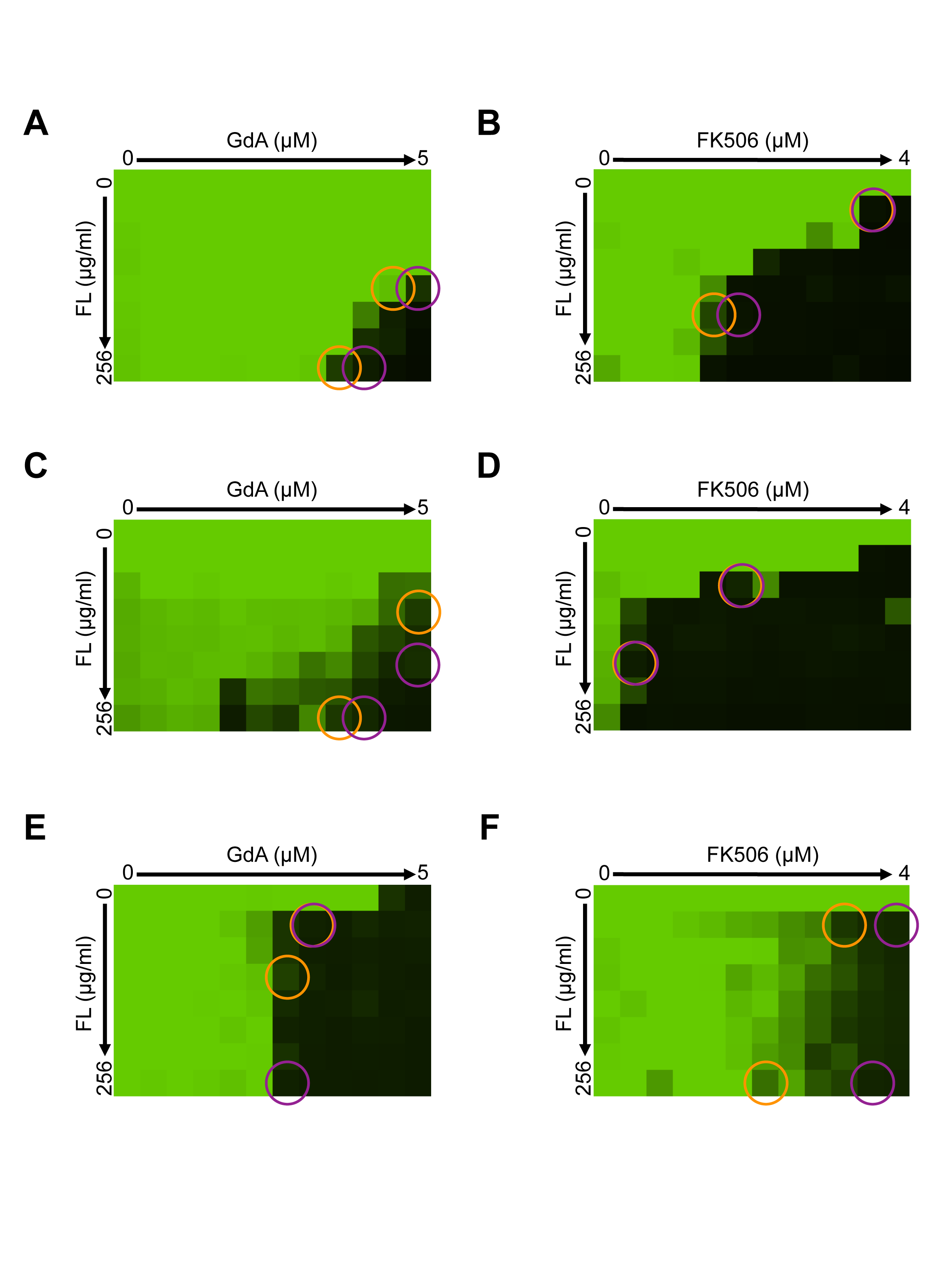

Supplement: Figure S2 — Dose response matrices were used to select concentrations for the evolution experiment. Dose response matrices for parental strains: (A, B) Sc erg3 W148*; (C, D) Sc erg3Δ; and (E, F) Ca erg3Δ/erg3Δ. Monitoring growth in the concentration gradients of azole and geldanamycin (A, C, and E) or azole and FK506 (B, D, and F) was used to determine the concentrations of the drug combinations for the experimental evolution study in order to reduce growth relative to a no-drug control, but minimize the probability of extinction. Evolution experiments were initiated with the drug combinations indicated by the orange circles (24-well plates) and purple circles (96-well plates). Resistance assays were performed as in Figure S1, with incubation for 3 days at 30°C. FL = fluconazole; and GdA = geldanamycin. (TIF) [file pgen.1003390.s002.tif]
