# Supplementary material for: Genetic and Genomic Architecture of the Evolution of Resistance to Antifungal Drug Combinations
Source: PLoS Genet. 2013 Apr 4;9(4):e1003390. doi: 10.1371/journal.pgen.1003390 (PMC3617151; doi:10.1371/journal.pgen.1003390)
Supplement: Table S1 — Mean coverage for whole genome sequenced strains. (DOCX) [file pgen.1003390.s003.docx]

**Table S1. Mean coverage whole-genome sequenced strains.**

| **Strain** | **Mean Coverage** |
| --- | --- |
| Sc-F-1 | 102 |
| Sc-G-13 | 71 |
| Ca-F-4 | 75 |
| Ca-F-5 | 176 |
| Ca-F-6 | 163 |
| Ca-F-7 | 179 |
| Ca-F-8 | 165 |
| Ca-F-9 | 204 |
