# Supplementary material for: Genetic and Genomic Architecture of the Evolution of Resistance to Antifungal Drug Combinations
Source: PLoS Genet. 2013 Apr 4;9(4):e1003390. doi: 10.1371/journal.pgen.1003390 (PMC3617151; doi:10.1371/journal.pgen.1003390)
Supplement: Table S2 — Number of high confidence single nucleotide variants (coding and non-coding). (DOCX) [file pgen.1003390.s004.docx]

**Table S2. Number of high confidence single nucleotide variants (SNVs)**

**(coding and non-coding).**

| **Strain** | **# of SNVs** |
| --- | --- |
| Sc-F-1 | 60 |
| Sc-G-13 | 135 |
| Ca-F-4 | 169 |
| Ca-F-5 | 23 |
| Ca-F-6 | 23 |
| Ca-F-7 | 20 |
| Ca-F-8 | 21 |
| Ca-F-9 | 9 |
