# Supplementary material for: Genetic and Genomic Architecture of the Evolution of Resistance to Antifungal Drug Combinations
Source: PLoS Genet. 2013 Apr 4;9(4):e1003390. doi: 10.1371/journal.pgen.1003390 (PMC3617151; doi:10.1371/journal.pgen.1003390)
Supplement: Table S5 — Strains used in this study. (DOCX) [file pgen.1003390.s007.docx]

**Table S5. Strains used in this study.**

| **Strain name** | **Strain number** | **Species** | **Genotype** | **Source** |
| --- | --- | --- | --- | --- |
| *Sc erg3*^W148*^ | ScLC7 | *S. cerevisiae* | *ura3*::KAN, *ERG3*^W148*^ | [[3](#_ENREF_3)] |
| *Sc erg3∆* | ScLC10 | *S. cerevisiae* | As BY4741 (*his3∆1 leu2∆0 met15∆0 ura3∆0*); *erg3*::KAN | [[3](#_ENREF_3)] |
| *Ca erg3∆/erg3∆* | CaLC660 | *C. albicans* | *arg4/arg4 his1/his1* *URA3/ura3*::*imm434* *IRO1/iro1*::*imm434 CaTAR::HIS3 erg3::FRT/erg3::FRT* | [[4](#_ENREF_4)] |
| Sc-F-1 | ScLC1367 | *S. cerevisiae* | As ScLC7, *MOT3* (G265*) | This study |
| Sc-F-2 | ScLC1441 | *S. cerevisiae* | As ScLC7, *FKS1* (dupG53 – D61) | This study |
| Sc-F-3 | ScLC1443 | *S. cerevisiae* | As ScLC7, *FKS1* (V108F) | This study |
| Ca-F-4 | CaLC1370 | *C. albicans* | As CaLC660 | This study |
| Ca-F-5 | CaLC1371 | *C. albicans* | As CaLC660 | This study |
| Ca-F-6 | CaLC1372 | *C. albicans* | As CaLC660 | This study |
| Ca-F-7 | CaLC1373 | *C. albicans* | As CaLC660 | This study |
| Ca-F-8 | CaLC1374 | *C. albicans* | As CaLC660 | This study |
| Ca-F-9 | CaLC1503 | *C. albicans* | As CaLC660 | This study |
| Ca-G-10 | CaLC1486 | *C. albicans* | As CaLC660, *HSP90* (D91Y) | This study |
| Sc-G-11 | ScLC1538 | *S. cerevisiae* | As ScLC10 | This study |
| Sc-G-12 | ScLC1539 | *S. cerevisiae* | As ScLC7, *HSC82* (K385*) | This study |
| Sc-G-13 | ScLC1540 | *S. cerevisiae* | As ScLC10, *PDR1* (P865R) | This study |
| Sc-G-14 | ScLC1541 | *S. cerevisiae* | As ScLC10, *HSC82* (I117N) | This study |
| *Sc erg3∆ pdr1∆* | ScLC485 | *S. cerevisiae* | his3∆0 leu2∆0 met15∆0 ura3∆0 lys2∆0 *erg3*::KAN *pdr1*::KAN |  |
| Sc-F-1 *cnb1∆* | ScLC1437 | *S. cerevisiae* | As ScLC1367, *cnb1*::NAT | This study |
| *Sc erg3 cnb1∆* | ScLC1439 | *S. cerevisiae* | As ScLC7, *cnb1*::NAT | This study |
| Sc-F-2 *cnb1∆* | ScLC1454 | *S. cerevisiae* | As ScLC1441, *cnb1*::NAT | This study |
| Sc-F-3 *cnb1∆* | ScLC1456 | *S. cerevisiae* | As ScLC1443, *cnb1*::NAT | This study |
| Sc-F-3 *fpr1∆* | ScLC1569 | *S. cerevisiae* | As ScLC1443, *fpr1*::HYGB | This study |
| *Sc erg3* ^W148*^ *fpr1∆* | ScLC1570 | *S. cerevisiae* | As ScLC7, *fpr1*::HYGB | This study |
| Sc-F-3 *fpr1∆* | ScLC1584 | *S. cerevisiae* | As ScLC1569, pLC564 (*URA3*) | This study |
| Sc-F-3 *fpr1∆* | ScLC1585 | *S. cerevisiae* | As ScLC1570, pLC564 (*URA3*) | This study |
| Sc-F-3 *fpr1∆* | ScLC1598 | *S. cerevisiae* | As ScLC1569, pLC565 (*URA3*) | This study |
| Sc-F-3 *fpr1∆* | ScLC1599 | *S. cerevisiae* | As ScLC1570, pLC565 (*URA3*) | This study |
| Sc-G-12 *hsc82∆* | ScLC1650 | *S. cerevisiae* | As ScLC1539, *hsc82*::HYGB | This study |
| Sc-G-14 *hsc82∆* | ScLC1652 | *S. cerevisiae* | As ScLC1541, *hsc82*::HYGB | This study |
| *Sc erg3∆ hsc82∆* | ScLC1658 | *S. cerevisiae* | *erg3*::natR *hsc82*::kanR can1::*MFA1*pr-*HIS3* *lyp-*1 *leu2∆0 ura3∆0 met15∆0* | [[5](#_ENREF_5)] |
| Sc-F-2 *fpr1∆* | ScLC1879 | *S. cerevisiae* | As ScLC1441, *fpr1*::HYGB | This study |
| Sc-G-14 *hsc82∆* + p*HSC82* | ScLC2024 | *S. cerevisiae* | As Sc-G-14, pLC28 (*LEU2*) | This study |
| Sc-G-14 *hsc82∆* + p*HSC82*^I117N^ | ScLC2025 | *S. cerevisiae* | As ScLC1541, pLC636 (*LEU2*) | This study |
| *Sc erg3∆* *hsc82∆* + p*HSC82* | ScLC2026 | *S. cerevisiae* | As ScLC1658, pLC28 (*LEU2*) | This study |
| *Sc erg3∆* *hsc82∆* + p*HSC82*^I117N^ | ScLC2027 | *S. cerevisiae* | As ScLC1658, pLC636 (*LEU2*) | This study |
| Sc-F-2 *fpr1∆* + p*FPR1* | ScLC2126 | *S. cerevisiae* | As ScLC1879, pLC564 (*URA3*) | This study |
| *Sc erg3* ^W148*^ *fpr1∆* + p*FPR1*^dupG53 –^ ^D61^ | ScLC2127 | *S. cerevisiae* | As ScLC1570, pLC653 (*URA3*) | This study |
| Sc-F-2 *fpr1∆* + p*FPR1*^dupG53 –^ ^D61^ | ScLC2128 | *S. cerevisiae* | As ScLC1879, pLC564 (*URA3*) | This study |
| Sc-G-13 *pdr1∆* | ScLC2134 | *S. cerevisiae* | As ScLC1540, *pdr1*::HYGB | This study |
| *Sc erg3* ^W148*^ *hsc82∆* | ScLC2139 | *S. cerevisiae* | As ScLC7, *hsc82*::HYGB | This study |
| Ca-G-10 *HSP90/HSP90* | CaLC2293 | *C. albicans* | As CaLC1486, pLC455 | This study |
| Ca-G-10 *HSP90/HSP90* | CaLC2294 | *C. albicans* | As CaLC1486, pLC455 | This study |
| *Ca erg3/erg3* *HSP90/HSP90^D91Y^* | CaLC2339 | *C. albicans* | As CaLC660, pLC700 | This study |
| *Ca erg3/erg3* *HSP90/HSP90^D91Y^* | CaLC2340 | *C. albicans* | As CaLC660, pLC700 | This study |
| *Sc erg3* ^W148*^ *mot3∆* | ScLC2455 | *S. cerevisiae* | As ScLC7, *mot3*::HYGB | This study |
| Sc-F-1 *mot3∆* | ScLC2457 | *S. cerevisiae* | As ScLC1367, *mot3*::HYGB | This study |
