# Supplementary material for: Genetic and Genomic Architecture of the Evolution of Resistance to Antifungal Drug Combinations
Source: PLoS Genet. 2013 Apr 4;9(4):e1003390. doi: 10.1371/journal.pgen.1003390 (PMC3617151; doi:10.1371/journal.pgen.1003390)
Supplement: Table S6 — Plasmids used in this study. (DOCX) [file pgen.1003390.s008.docx]

**Table S5. Plasmids used in this study.**

| Plasmid | Description | Source |
| --- | --- | --- |
| pLC28 | *GPD1p-HSC82-CYC1t (LEU2)* (p415GPDHSC82) | Leah Cowen |
| pLC455 | *C. albicans* *HSP90* complementation vector | [[1](#_ENREF_1)] |
| pLC537 | *S. cerevisiae CNB1* knock-out construct | This study |
| pLC564 | *GPD1p-FPR1-CYC1t (URA3)* | This study |
| pLC565 | *GPD1p-FPR1^V108F^-CYC1t (URA3)* | This study |
| pLC636 | *GPD1p-HSC82*^I117N^*-CYC1t (LEU2)* | This study |
| pLC653 | *GPD1p-FPR1^dupG53-D61^-CYC1t (URA3)* | This study |
| pLC661 | *GPD1p-PDR1-CYC1t (URA3)* | This study |
| pLC700 | pLC455, *HSP90*^D91Y^ | This study |
