# Supplementary material for: Genetic and Genomic Architecture of the Evolution of Resistance to Antifungal Drug Combinations
Source: PLoS Genet. 2013 Apr 4;9(4):e1003390. doi: 10.1371/journal.pgen.1003390 (PMC3617151; doi:10.1371/journal.pgen.1003390)
Supplement: Table S7 — Oligonucleotides used in this study. (DOCX) [file pgen.1003390.s009.docx]

**Table S7. Oligonucleotides used in this study.**

­­­­­­­­­­­­­­Primer Sequence (5’ 🡪 3’)

oLC1 GGTTTGTGAACAGATTGACAAGC

oLC2 AGGCTGGTCAAGTCTGAAGC

oLC5 GTTGTACGTTCGTCGTGTC

oLC9 GTGTTCTGGCATTCTAGTAACG

oLC11 CAGCAGATAGAGCTTCCATG

oLC59 CAGAAACTTCTCGACAGACG

oLC60 CTCGTCCGAGGGCAAAGG

oLC65 GCATATTGCACTAACGTGAGG

oLC76 CCTATCAAACAATCCTGCC

oLC101 CTGCAGCGAGGAGCCGTAAT

oLC146 TGATTTTGATGACGAGCGTAAT

oLC200 GAAATTTCTCCATCTTCC

oLC274 CTGTCAAGGAGGGTATTCTGG

oLC275 AAAGTCAAAGTTCCAAGGGG

oLC311 ACCCTAAATGGAGTTTCTTTTCTTG

oLC312 TTCACTTTTCATTTTCTTCCAGAAC

oLC332 TTGCGGGCCCAACAAGATCAATACCTTACTACC

oLC333 ATAAGAATGCGGCCGCGATCTATCTTGAAACTCAGCG

oLC334 GGCACCGCGGCTCTGATCGTATACCTATCC

oLC355 ATCAAAGAGCAAAATTGGC

oLC530 GTTACTTGATGATATTAAGCACGGG

oLC659 AGATGCGAAGTTAAGTGCGC

oLC805 CGGTAGGTATTGATTGTAATTCTG

oLC867 TTGCGGGCCCGTATTGAGACAGAAGAAGTG

oLC885 CCTTGTCTAGTGCTGAACGTGC

oLC1049 TTTTAAAGCTTCCAGCTTCCTATTT

oLC1168 GGGGTCGACCCCATTTTAAGAAATAAAAATGCTTCT

ATGG

oLC1169 GGGAAGCTTAACTGACTACAAGACATACC

oLC1170 TTAGGGGAAGATATACTAGC

oLC1171 GGGGAGCTCTAATACGCGATATTGTTTTTTAAGG

oLC1172 GGGGAATTCGATAGTCATCGGGTTTAACG

oLC1173 ATCAAAGATGGTCTGTCTCC

oLC1371 CTATTTCAATTAAGGCTCAG

oLC1388 GAAATCCGCGTGCACGAGGT

oLC1389 CCGATCTTAGCCAGACGAGC

oLC1437 GTCATCGCCACAACTTGAGC

oLC1438 GAAGTAATTGAAGGTAACGTC

oLC1439 TGACCTTCAACAATTCGACG

oLC1440 CATTGGATCCATGTCTGAAGTAATTGAAGG

oLC1446 TTGCGTCGACTTAGTTGACCTTCAACAATTCG

oLC1517 CAGACCCTGAATACAAGGAA

oLC1518 CGTATGCTTCTCCTTGTCTA

oLC1733 GTTAGTCTTTTTTTTAGTTTTAAAACACCAGAACTTAGTTT

CGACGGATGAATCATTCTGAAATATGGC

oLC1734 CTCGAGGTCGACGGTATCGATAAGCTTGATATCGAATTCC

TGCAGCCCGGGAGATTAATCAACTTCTTCC

oLC2146 TTGTTGAATGATAGCTACGG

oLC2438 ACTAATAGGCAACAGTAGGC

oLC2440 AGTAATGGCTTATGTATGAAGCAGG

oLC2530 CTGCGATCCGCTTTATTACG

oLC2531 GTGTATTGCACTCGGGATAG

oLC2533 GGACAGTTCATCAATGTGGG

________________________________________________________________________
