# Supplementary material for: Genetic and Genomic Architecture of the Evolution of Resistance to Antifungal Drug Combinations
Source: PLoS Genet. 2013 Apr 4;9(4):e1003390. doi: 10.1371/journal.pgen.1003390 (PMC3617151; doi:10.1371/journal.pgen.1003390)
Supplement: Text S1 — Supporting Materials and Methods. (DOC) [file pgen.1003390.s011.doc]

**Text S1**

**Supporting Materials and Methods**

***Strain construction***

***Sc erg3∆ pdr1∆*(ScLC485):** The BY4742-derived strain *erg3∆pdr1∆ pdr3∆* MATα was mated with the BY4741-derived strain *pdr1∆* MATa. Diploids were sporulated in liquid medium and tetrads were dissected. Meiotic progeny were tested for *pdr1Δ* by PCR using primers oLC311/101 and *erg3∆* using primers oLC65/146. The presence of *PDR3* was verified by PCR with primers oLC312/101 (absence of a band).

**Sc-F-1 *cnb1∆* (ScLC1437):** The plasmid pLC537 was digested with NotI to liberate the NAT-FLIP cassette and was transformed into Sc-F-1. For NAT-resistant transformants, proper integration was tested by PCR with primer pairs oLC1170/oLC274 and oLC659/oLC1173.

***Sc erg3*^W148*^ *cnb1∆* (ScLC1439):** The plasmid pLC537 was digested with NotI to liberate the NAT-FLIP cassette and was transformed into *Sc erg3*^W148*^. For NAT-resistant transformants, proper integration was tested by PCR with primer pairs oLC1170/oLC274 and oLC659/oLC1173.

**Sc-F-2 *cnb1∆* (ScLC1454):** The plasmid pLC537 was digested with NotI to liberate the NAT-FLIP cassette and was transformed into Sc-F-2. For NAT-resistant transformants, proper integration was tested by PCR with primer pairs oLC1170/274 and oLC659/1173.

**Sc-F-3 *cnb1∆* (ScLC1456):** The plasmid pLC537 was digested with NotI to liberate the NAT-FLIP cassette and was transformed into Sc-F-3. For NAT-resistant transformants, proper integration was tested by PCR with primer pairs oLC1170/274 and oLC659/1173.

**Sc-F-3 *fpr1∆* (ScLC1569):** The hygromycin B resistance cassette was amplified from a BY4741 strain with *fpr1::*KAN-MX by PCR with primers oLC530/1371. The resulting amplicon has ~300bp of sequence homologous to *FPR1* upstream and ~80bp of sequence homologous to *FPR1* downstream. The amplicon was transformed into Sc-F-3 and plated on YPD + hygromycin B. Hygromycin B-resistant transformants were tested by PCR with primers oLC1517/1439 and oLC1518/1438 for absence of *FPR1*. Integration of hygromycin B cassette in the *FPR1* locus was tested for by PCR with primer pairs oLC1517/59 and oLC1518/60.

***Sc erg3*^W148*^ *fpr1∆* (ScLC1570):** The hygromycin B resistance cassette was amplified from a BY4741 strain *fpr1::*KAN-MX by PCR using primers oLC530/1371. The resulting amplicon has ~300bp of sequence homologous to *FPR1* upstream and ~80bp of sequence homologous to *FPR1* downstream. The amplicon was transformed into *Sc erg3*^W148*^ and plated on YPD + hygromycin B. Hygromycin B-resistant transformants were tested by PCR with primer pairs oLC1517/1439 and oLC1518/1438 for absence of *FPR1*. Integration of hygromycin B cassette in the *FPR1* locus was tested for by PCR with primer pairs oLC1517/59 and oLC1518/60.

**Sc-F-3 *fpr1∆* + p*FPR1* (ScLC1584):** Sc-F-3 *fpr1∆* was transformed with pLC564 and plated on synthetic defined medium lacking uracil. Colonies carrying pLC564 were PCR confirmed using primers oLC805/1439.

***Sc erg3*^W148*^ *fpr1∆* + p*FPR1* (ScLC1585):** *Sc erg3*^W148*^ *fpr1∆* was transformed with pLC564 and plated on synthetic defined medium lacking uracil. Colonies carrying pLC564 were PCR confirmed using primers oLC805/1439.

**Sc-F-3 *fpr1∆* + p*FPR1*^V108F^ (ScLC1598):** Sc-F-3 *fpr1∆* was transformed with pLC565 and plated on synthetic defined medium lacking uracil. Colonies carrying pLC564 were PCR confirmed using primers oLC805/1439.

***Sc erg3*^W148*^ *fpr1∆* + p*FPR1*^V108F^ (ScLC1599):** *Sc erg3*^W148*^ *fpr1∆* was transformed with pLC565 and plated on synthetic defined medium lacking uracil. Colonies carrying pLC564 were PCR confirmed using primers oLC805/1439.

**Sc-G-12 *hsc82∆* (ScLC1650):** The hygromycin B resistance cassette was amplified from a BY4741 strain with *hsc82::*HYGB with up- and downstream sequence homologous to *HSC82* by PCR using primers oLC1/2. The amplicon was transformed into Sc-G-12 and plated on YPD + hygromycin B. Hygromycin B-resistant transformants were tested for absence of *HSC82* by PCR with primers oLC1/11 and for the presence the HYGB marker integrated at the *HSC82* locus by PCR with primers oLC2/60.

**Sc-G-14 *hsc82∆* (ScLC1652):** The hygromycin B resistance cassette was amplified from a BY4741 strain with *hsc82::*HYGB with up- and downstream sequence homologous to *HSC82* by PCR with primers oLC1/2. The PCR fragment was transformed into Sc-G-14 and plated on YPD + hygromycin B. Hygromycin B-resistant transformants were tested for absence of *HSC82* by PCR with primers oLC1/11 and for the presence HYGB at the *HSC82* locus by PCR with primers oLC2/60.

***Sc erg3∆* *hsc82∆* (ScLC1658):** This strain was acquired from the SGA *erg3****∆***double deletion library and was verified by PCR for the absence of *ERG3* (primers oLC76/1049) and for the absence of *HSC82* (primers oLC1/oLC11).

**Sc-F-2 *fpr1∆* (ScLC1879):** The hygromycin B resistance cassette was amplified from a BY4741 strain with *fpr1::*KAN-MX by PCR using primers oLC530/1518. The resulting amplicon was transformed into Sc-F-2 and plated on YPD + hygromycin B. Hygromycin B-resistant transformants were tested for proper integration at the *FPR1* locus by PCR with primers oLC1517/59 and for absence of *FPR1* with primers oLC1437/1517.

**Sc-G-14 *hsc82∆ +* p*HSC82* (ScLC2024):** Sc-G-14 *hsc82∆* was transformed with pLC28 and plated on synthetic defined medium with histidine, methionine, and uracil, but lacking leucine. Colonies carrying pLC28 were confirmed by PCR using primers oLC805/11.

**Sc-G-14 *hsc82∆ +* p*HSC82*^I117N^ (ScLC2025):** Sc-G-14 *hsc82∆* was transformed with pLC636 and plated on SD + HIS + MET + URA. Colonies carrying pLC636 were PCR confirmed using oLC805/11.

***Sc erg3∆* *hsc82∆ +* p*HSC82* (ScLC2026):** *Sc erg3****∆*** *hsc82∆* was transformed with pLC28 and plated on synthetic defined medium with histidine, methionine, and uracil, but lacking leucine. Colonies carrying pLC28 were confirmed by PCR using primers oLC805/11.

***Sc erg3∆* *hsc82∆ +* p*HSC82*^I117N^ (ScLC2027):** *Sc erg3****∆*** *hsc82∆* was transformed with pLC636 and plated on synthetic defined medium with histidine, methionine, and uracil, but lacking leucine. Colonies carrying pLC636 were confirmed by PCR using primers oLC805/11.

**Sc-F-2 *fpr1∆* + p*FPR1* (ScLC2126):** Sc-F-2 *fpr1∆* was transformed with pLC564 and plated on synthetic defined medium lacking uracil. Colonies carrying pLC564 were confirmed by PCR using primers oLC805/1439.

***Sc erg3*^W148*^ *fpr1∆* + p*FPR1*^dupG53 – D61^ (ScLC2127):** *Sc erg3***^W148*^** *fpr1∆* was transformed with pLC653 and plated on synthetic defined medium lacking uracil. Colonies carrying pLC653 were confirmed by PCR using primers oLC805/1439.

**Sc-F-2 *fpr1∆* + p*FPR1*^dupG53 – D61^ (ScLC2128):** Sc-F-2 *fpr1∆* was transformed with pLC653 and plated on synthetic defined medium lacking uracil. Colonies carrying pLC653 were confirmed by PCR using primers oLC805/1439.

**Sc-G-13 *pdr1∆* (ScLC2134):** The hygromycin B resistance cassette was amplified from a BY4741 strain with *pdr1::*KAN by PCR using primers oLC311/2146. The PCR fragment was transformed into Sc-G-13 and plated on YPD + hygromycin B. Hygromycin B-resistant transformants were tested for proper integration of the hygromycin B marker by PCR with primers oLC1388/1389 and for the absence of *PDR1* with primers oLC311/885.

***Sc erg3*^W148*^ *hsc82∆* (ScLC2139):** The hygromycin B resistance cassette was amplified from a BY4741 strain with *hsc82::*KAN by PCR using primers oLC1/2. The resulting amplicon was transformed into *Sc erg3*^W148*^ and plated on YPD + hygromycin B. Hygromycin B-resistant transformants were tested for proper integration of the hygromycin B marker by PCR with primers oLC1388/1389 and for the absence of *HSC82* with primers oLC5/9.

**Ca-G-10 *HSP90/HSP90* (CaLC2293):** The plasmid pLC455 was digested with BssHII to liberate the *HSP90* allele replacement cassette and was transformed into Ca-G-10. For NAT-resistant transformants, proper integration was tested by PCR with primers oLC355/274. The *SAP2* promoter was induced to drive expression of FLP recombinase to excise the NAT marker cassette. This is the first transformant.

**Ca-G-10 *HSP90/HSP90* (CaLC2294):** The plasmid pLC455 was digested with BssHII to liberate the *HSP90* allele replacement cassette and was transformed into Ca-G-10. For NAT-resistant transformants, proper integration was tested by PCR with primers oLC355/274. The *SAP2* promoter was induced to drive expression of FLP recombinase to excise the NAT marker cassette. This is the second transformant.

***Ca erg3∆/erg3∆ HSP90/HSP90*^D91Y^** **(CaLC2339):** The plasmid pLC701 was digested with BssHII to liberate the *HSP90*^D91Y^ allele replacement cassette and was transformed into *Ca erg3∆/erg3∆*. For NAT-resistant transformants, proper integration was tested by PCR with primers oLC355/oLC274. The *SAP2* promoter was induced to drive expression of FLP recombinase to excise the NAT marker cassette. Presence of the *HSP90*^D91Y^ allele was verified by amplifying the N-terminal region of *HSP90* and sequencing over the mutation. This is the first transformant.

***Ca erg3∆/erg3∆ HSP90/HSP90*^D91Y^** **(CaLC2340):** The plasmid pLC701 was digested with BssHII to liberate the *HSP90*^D91Y^ allele replacement cassette and was transformed into *Ca erg3∆/erg3∆*. For NAT-resistant transformants, proper integration was tested by PCR with primers oLC355/oLC274. The *SAP2* promoter was induced to drive expression of FLP recombinase to excise the NAT marker cassette. Presence of the *HSP90*^D91Y^ allele was verified by amplifying the N-terminal region of *HSP90* and sequencing over the mutation. This is the second transformant.

***Sc erg3*^W148*^ *mot3∆* (ScLC2455):** The hygromycin B resistance cassette was amplified from a BY4741 strain with *mot3::*KAN by PCR using primers oLC2530/2531. The resulting amplicon was transformed into *Sc erg3*^W148*^ and plated on YPD + hygromycin B. Hygromycin B-resistant transformants were tested for proper integration of the marker at the *MOT3* locus by PCR with primers oLC2438/2440 and oLC2533/60.

**Sc-F-1 *mot3∆* (ScLC2457):** The hygromycin B resistance cassette was amplified from a BY4741 strain with *mot3*::KAN using oLC2530/2531. The PCR fragment was transformed into Sc-F-1 and plated on YPD + hygromycin B. Hygromycin B-resistant transformants were tested for proper integration of the marker at the *MOT3* locus PCR with primers oLC2438/2440 and oLC2533/60.

***Plasmid construction***

**pLC28:** *HSC82* was placed under the control of the *GPD1* promoter on a *LEU2* marked centromeric plasmid. *HSC82* was cloned into p415GPD using BamHI and SpeI.

**pLC537:** This is a construct to knock out *CNB1* in *Saccharomyces cerevisiae* using a NAT marker flanked by 400-500 bp of sequence homologous to the upstream and downstream regions flanking the coding sequence of *CNB1*. The upstream region of *CNB1* was amplified by PCR using primers oLC1168/oLC1169 and the downstream region was amplified using primers oLC1171/oLC1172. The amplicons were cloned into pLC1. pLC1 and the upstream sequence were sequentially digested with SalI and HindIII, then ligated. The presence of the insert was tested by PCR with primers oLC1169/274. pLC1 with the sequence upstream of *CNB1* and the downstream sequence were digested with SacI and EcoRI, then ligated. The presence of the downstream insert was tested by PCR with primers oLC1172/659. The downstream sequence homology begins 46 bp downstream of the *CNB1* stop codon, thus *CNB1* + 46bp downstream are knocked out using this construct. The knock-out construct can be liberated by digestion with NotI.

**pLC564:** *FPR1* was amplified from *Sc erg3* genomic DNA using oLC1440/1446. The PCR product and pLC138 (p416GPD) were digested with BamHI and SalI, then ligated. The presence of the insert was verified by PCR with primers oLC805/1439.

**pLC565:** *FPR1*^V108F^ was amplified from Sc-F-3 genomic DNA using oLC1440/1446. The PCR product and pLC138 (p416GPD) were digested with BamHI and SalI, then ligated. The presence of the insert was verified by PCR with primers oLC805/1439.

**pLC636:** *HSC82*^I117N^ was amplified from Sc-G-14 genomic DNA using oLC1733/1734. The PCR product and pLC136 (p415GPD) were digested with BamHI and SpeI, then ligated. The presence of the insert was verified by PCR with primers oLC805/11.

**pLC653:** *FPR1*^dupG53 –^ ^D61^ was amplified from Sc-F-2 genomic DNA using oLC2045/2046. The PCR product and pLC138 (p416GPD) were digested with BamHI and SpeI, then ligated. The presence of the insert was verified by PCR with primers oLC805/1439.

**pLC701:** *HSP90*^D91Y^ (and sequence upstream) was amplified from Ca-G-10 genomic DNA by PCR using primers oLC332/867. pLC49 and Ca-G-10 oLC332/867 were digested with ApaI, CIP treated and ligated. The presence and orientation of the insert was verified by PCR with primers oLC275/oLC200. Downstream of *HSP90* was amplified by PCR with primers oLC333/334. pLC49 with Ca-G-10 oLC332/867 and CaLC1486 oLC333/334 were double digested with NotI-HF and SacII and ligated. The presence of the insert was verified by PCR with primers oLC274/334. The allele replacement cassette is liberated by digestion with BssHII.

**References**

1. Cowen LE, Singh SD, Kohler JR, Collins C, Zaas AK, et al. (2009) Harnessing Hsp90 function as a powerful, broadly effective therapeutic strategy for fungal infectious disease. Proc Natl Acad Sci U S A 106: 2818-2823.

2. Shen J, Cowen LE, Griffin AM, Chan L, Kohler JR (2008) The *Candida albicans* pescadillo homolog is required for normal hypha-to-yeast morphogenesis and yeast proliferation. Proc Natl Acad Sci U S A 105: 20918-20923.

3. Cowen LE, Lindquist S (2005) Hsp90 potentiates the rapid evolution of new traits: drug resistance in diverse fungi. Science 309: 2185-2189.

4. Robbins N, Collins C, Morhayim J, Cowen LE (2010) Metabolic control of antifungal drug resistance. Fungal Genet Biol 47: 81-93.

5. Tong AH, Lesage G, Bader GD, Ding H, Xu H, et al. (2004) Global mapping of the yeast genetic interaction network. Science 303: 808-813.
